# Functional Characterization and Structural Insights Into Stereoselectivity of Pulegone Reductase in Menthol Biosynthesis

**DOI:** 10.3389/fpls.2021.780970

**Published:** 2021-11-30

**Authors:** Chanchan Liu, Qiyu Gao, Zhuo Shang, Jian Liu, Siwei Zhou, Jingjie Dang, Licheng Liu, Iris Lange, Narayanan Srividya, B. Markus Lange, Qinan Wu, Wei Lin

**Affiliations:** ^1^School of Pharmacy, Nanjing University of Chinese Medicine, Nanjing, China; ^2^Jiangsu Collaborative Innovation Center of Chinese Medicinal Resources Industrialization, Nanjing, China; ^3^Department of Pathogen Biology, School of Medicine and Holistic Integrative Medicine, Nanjing University of Chinese Medicine, Nanjing, China; ^4^CAS Key Laboratory of Quantitative Engineering Biology, Guangdong Provincial Key Laboratory of Synthetic Genomics and Shenzhen Key Laboratory of Synthetic Genomics, Shenzhen Institute of Synthetic Biology, Shenzhen Institutes of Advanced Technology, Chinese Academy of Sciences, Beijing, China; ^5^Institute of Biological Chemistry and M.J. Murdock Metabolomics Laboratory, Washington State University, Pullman, WA, United States; ^6^State Key Laboratory of Natural Medicines, China Pharmaceutical University, Nanjing, China

**Keywords:** pulegone reductase, stereoselectivity, molecular dynamics simulations, menthol biosynthesis, *Mentha piperita*

## Abstract

Monoterpenoids are the main components of plant essential oils and the active components of some traditional Chinese medicinal herbs like *Mentha haplocalyx* Briq*., Nepeta tenuifolia* Briq*., Perilla frutescens* (L.) Britt *and Pogostemin cablin* (Blanco) Benth. Pulegone reductase is the key enzyme in the biosynthesis of menthol and is required for the stereoselective reduction of the Δ^2,8^ double bond of pulegone to produce the major intermediate menthone, thus determining the stereochemistry of menthol. However, the structural basis and mechanism underlying the stereoselectivity of pulegone reductase remain poorly understood. In this study, we characterized a novel (−)-pulegone reductase from *Nepeta tenuifolia* (*Nt*PR), which can catalyze (−)-pulegone to (+)-menthone and (−)-isomenthone through our RNA-seq, bioinformatic analysis in combination with *in vitro* enzyme activity assay, and determined the structure of (+)-pulegone reductase from *M. piperita* (*Mp*PR) by using X-ray crystallography, molecular modeling and docking, site-directed mutagenesis, molecular dynamics simulations, and biochemical analysis. We identified and validated the critical residues in the crystal structure of *Mp*PR involved in the binding of the substrate pulegone. We also further identified that residues Leu56, Val282, and Val284 determine the stereoselectivity of the substrate pulegone, and mainly contributes to the product stereoselectivity. This work not only provides a starting point for the understanding of stereoselectivity of pulegone reductases, but also offers a basis for the engineering of menthone/menthol biosynthetic enzymes to achieve high-titer, industrial-scale production of enantiomerically pure products.

## Introduction

Terpenoids are a large and structurally diverse group of natural products widely distributed in plants, microorganisms, and insects. More than 55,000 terpenoids have been identified so far, and the majority of them display diverse biological activities (Christianson, 2008). For example, some plant terpenoids function as chemical defense agents against predation and pathogens (Hijaz et al., 2016; Mahizan et al., 2019), and some play regulatory roles in the interactions with other plants and environment (Abbas et al., 2017). Among them, monoterpenoids are a type of terpenoids composed of two isoprene units and are widely distributed in plants with great therapeutic potential, such as traditional Chinese medicinal herbs like *Mentha haplocalyx* Briq (Duan et al., 2015), *Nepeta tenuifolia* Briq (Liu et al., 2018), *Perilla frutescens* (L.) Britt (Zhou et al., 2021), and *Pogostemin cablin* (Blanco) Benth (Wojtunik-Kulesza et al., 2019). As one of the representative monoterpenoids, menthol and its biosynthetic pathway as well as related biosynthetic enzymes have been identified (Gao et al., 2020). Briefly, the C_10_ menthol skeleton is formed by the condensation of isopentenyl pyrophosphate (IPP) and dimethylallyl pyrophosphate (DMAPP) (Gao et al., 2020), which are obtained from the 2C-methyl-D-erythritol-4-phosphate (MEP) pathway or the mevalonic acid (MVA) pathway in plant cells, followed by cyclization catalyzed by (−)-limonene synthase (LS). Subsequently, the C_10_ skeleton is further modified by a series of tailoring enzymes (e.g., monooxygenase, dehydrogenase, reductase, and isomerase) to generate (−)-menthol and the diastereomer (+)-isomenthol (Supplementary Figure 1A).

(−)-Menthone, the second most abundant monoterpenoid in peppermint essential oil, is a critical intermediate in menthol biosynthesis (Lange, 2015; Chen et al., 2019). In *Mentha piperita*, (−)-menthone is directly synthesized from (+)-pulegone through the reduction of C2–C8 alkene double bond catalyzed by (+)-pulegone reductase (EC number 1.3.1.81). Pulegone reductase is a double bond reductase (DBR) which belongs to the NADPH-dependent, medium-chain dehydrogenase/reductase (MDR) superfamily. Some DBRs in the MDR superfamily proteins have become important biotechnological tools for asymmetric synthesis due to their high stereoselectivity in the alkene double bond reduction (Currin et al., 2018). It has been reported that the reduction reaction proceeds through stereoselective transfer of a hydride from NADPH to the carbon of the substrate (Mcconkey et al., 2000; Ringer et al., 2003; Zebec et al., 2016; Paramasivan and Mutturi, 2017; Cheallaigh et al., 2018; Currin et al., 2018; Liu et al., 2018; Bergman et al., 2019; Wojtunik-Kulesza et al., 2019). In recent years, genes and enzymes accounting for double bond reduction in plant secondary metabolites have been identified through biochemical and structural studies. For instance, *Arabidopsis thaliana* L. homolog DBR (*At*DBR, UniProt ID: Q39172) reduces the C2-C8 double bond of *p*-coumaryl- and coniferyl-aldehydes (Youn et al., 2006). The *Nicotiana tabacum* L. DBR (*Nt*DBR, UniProt ID: Q9SLN8) has been shown to be active toward a variety of α,β-unsaturated alkenes (Mansell et al., 2013), such as (−)-cinnamaldehyde and 1-nitrocyclohexene. In addition, *Rubus idaeus* L. raspberry DBR (*Ri*DBR, UniProt ID: G1FCG0) catalyzes the reduction of 4-hydroxybenzalacetone and 3-methoxy-4-hydroxybenzalacetone to raspberry ketone and zingerone, respectively (Simon et al., 2017). A DBR from *Malus domestica* L. (*Md*DBR, UniProt ID: A0A5N5GUE7) has been isolated and characterized recently (Caliandro et al., 2021) and suggested to be involved in the biosynthesis of polyphenolic compounds (e.g., dihydrochalcones) that are beneficial in human diet. The structures of several plant derived DBRs (e.g., *At*DBR, *Nt*DBR, and *Ri*DBR) have been reported. However, stereoselectivity of the DBRs for substrates and products remains poorly understood.

In this report, we first performed functional characterization of pulegone reductases from *Nepeta tenuifolia* (*Nt*PR) and *Mentha piperita* (*Mp*PR, UniProt ID: Q6WAU0), from which we observed the two DBRs possess selectivity for both substrates and products in different diastereomeric forms. Phylogenetic analysis revealed both *Nt*PR and *Mp*PR belong to the MDR superfamily, but they are present in two different clades. To investigate the structural basis and underlying mechanism of the stereoselectivity, we performed molecular docking, molecular dynamic simulation, site-directed mutagenesis analysis, and biochemical assays based on the crystal structure of *Mp*PR. We uncovered the key residues in the pulegone binding pockets of *Nt*PR and *Mp*PR, and illustrated that the hydrophobic interactions and potential steric hindrances may mainly contribute to their stereoselectivity toward substrates and products. This study not only deepens our understanding on the substrate and product selectivity of alkene reductases, but also provides a structural and mechanistic basis for the engineering and directed evolution of pulegone reductases, enabling the production of enantiomerically pure end-product in industry.

## Materials and Methods

### Gene Cloning, Site-Directed Mutagenesis, Expression, and Purification of Pulegone Reductases

The (+)-pulegone reductase gene from *Mentha piperita* and (−)-pulegone reductase gene from *Nepeta tenuifolia* were cloned into the pET28a vector under control of the bacteriophage T7 gene promoters using *Nhe*I and *Hin*dIII, respectively. The (−)-pulegone reductase gene from *Agastache rugose* was cloned into the pET28a vector under control of the bacteriophage T7 gene promoters using *Bam*HI and *Sac*I. Each resulting plasmid was transformed into *E. coli* strain BL21(DE3) (Invitrogen). A single colony of the resulting transformants was used to inoculate 50 mL of LB broth containing 50 mg/mL kanamycin, and the culture was incubated at 37°C for 16 h with shaking. An aliquot (10 mL) was used to inoculate 1 L of LB broth containing 50 mg/mL kanamycin, followed by the incubation with agitation at 37°C, 180 rpm until the OD_600_ reached 0.8–1.0. Subsequently, the culture was supplemented with isopropyl-b-D-thiogalactoside to the final concentration of 0.5 mM for protein expression, and then the culture was incubated for 16 h at 20°C. Cells were harvested by centrifugation (5,000 × g; 15 min at 4°C), re-suspended in buffer A (10 mM Tris-HCl, pH 8.0, 200 mM NaCl, and 5% glycerol), and lysed using an EmulsiFlex-C5 cell disruptor (Avestin). The lysate was centrifuged (20,000 × g; 30 min at 4°C) and the cell debris was removed. The supernatant was loaded onto a 5 mL column of Ni^2+^-NTA-agarose (Qiagen) pre-equilibrated with buffer A. The column was washed with 10 × 5 mL buffer A containing 25 mM imidazole followed by elution with 30 mL buffer A containing 200 mM imidazole. The eluate was concentrated to ∼10 mg/mL and further purified by gel filtration chromatography on a HiLoad 16/60 Superdex 200 prep grade column (GE Healthcare) in 10 mM Tris-HCl, pH 8.0, 50 mM NaCl, 5 mM MgCl_2_, and 1 mM DTT. The peak fractions were collected and concentrated to the final concentration of 20 mg/mL in the same buffer using 30 kDa MWCO Amicon Ultra-15 centrifugal ultrafilters (EMD Millipore), and stored in aliquots at –80°C. The yields of the proteins were ∼20 mg/mL, and purities were ∼95%. Site-directed mutations were prepared using one step PCR method, and the mutated proteins were expressed and purified by following the same protocol as the wild-type proteins. High and low molecular weight (mass) calibration kits (GE Health-care) were used to calibrate the molecular mass of wild type *Mp*PR.

### *In vitro* Enzyme Activity Assays

The reduction reaction (0.4 mL) catalyzed by pulegone reductase was performed in buffer B (50 mM KH_2_PO_4_, 10% sorbitol, 1 mM DTT, pH 7.5), containing 20 μM substrate [(+)-pulegone (CAS No: 89-82-7) or (−)-pulegone (CAS No: 3391-90-0), Sigma], 10 mM NADPH tetrasodium salt hydrate (CAS No:2646-71-1, Sigma), 6 mM glucose-6-phosphate, 20 U glucose-6-phosphate dehydrogenase (Solarbio), and 30 μM *Mp*PR or 36 μM *Nt*PR. 0.2 mL of *n*-hexane was added on the top of the reaction solution. Reaction was carried out at 31°C for 1 h (*Mp*PR) and 16 h (*Nt*PR) with slowly stirring. The reaction was terminated by placing the reaction vial at –20°C for 2 h. The upper organic phase was transferred into a new 2 mL glass vial containing a conical glass insert and immediately analyzed by GC-MS and chiral GC. The negative control containing inactive enzyme was generated by heating the reconstitution mix for 15 min at 95°C.

GC-MS analysis was performed by Agilent 7890B/7000C, equipped with a HP 5MS capillary column (30 m × 0.25 mm; film thickness 0.25 μm). The programmed temperatures of the column were set as follows: 85°C for 4 min, 85–130°C at 5°C/min, 130°C for 2 min, 130–140°C at 5°C/min, 140°C for 3 min. Ion source temperature was set at 230°C. Electron ionization (EI) mass spectra were acquired over the mass range 50–500 Da at the energy of 70 eV. Chiral GC analysis was carried out using Agilent 8860, equipped with a CYCLODEX-B capillary column (30 m × 0.32 mm; film thickness 0.25 μM). The programmed temperatures of the column were set as follows: 80–95°C at 2°C/min, 95–110°C at 0.5°C/min. Injector temperature and carrier gas (Nitrogen) detector temperature were set at 250°C.Nitrogen was used as the carrier gas at a flow rate of 1 mL/min with an injection volume of 1 μL, no split.

### Measurement of Kinetic Parameters for the Wild-Type and Mutated *Mentha piperita* and *Nepeta tenuifolia*

The reduction reaction was initiated in a 400 μL solution containing 50 mM KH_2_PO_4_, 10% sorbitol, 1 mM DTT, 10 mM NADPH tetrasodium salt hydrate, 6 mM glucose-6-phosphate, 20 U glucose-6-phosphate dehydrogenase (Solarbio), together with the wild-type and mutated *Mp*PR, *Ar*PR, and *Nt*PR (Supplementary Table 1). Other conditions are the same as the *in vitro* enzyme catalysis assay. Quantitative analysis was performed by the comparison of the peak areas of products to the standards of known concentrations obtained from chiral GC analysis (Agilent 8860 equipped with a HP-5 capillary column; the column condition is the same as described above). All biotransformation reactions were performed in at least duplicates, and the results are averages of the data. The yields of menthone and iso-menthone at each concentration were calculated by the comparison of the peak areas of products to the standards of known concentrations. The kinetic parameters *K*_*m*_ and k_cat_ were calculated using Equation 1.


(1) $$V_{0}=V_{\text{max}}\left[S\right]/\left(k_{m}+\left[S\right]\right)$$

where *V*_0_ is the initial velocity, [*E*] is the enzyme concentration, [*S*] is the substrate concentration, *V*_max_ is the maximum velocity; *K*_*m*_ is the Michaelis constant and k_cat_ is calculated from *V*_max_/[*E*].

### Crystallization, Data Collection, and Structure Determination of *Mentha piperita*

Robotic crystallization trials were performed for *Mp*PR and *Nt*PR as well as co-crystallization with NADPH tetrasodium salt hydrate and (+)- or (−)-pulegone using a Griffin liquid handling system (Art Robbins Instruments), commercial screening solutions (Emerald Biosystems, Hampton Research, and Qiagen), and the sitting-drop vapor-diffusion technique (drop: 0.2 μL protein plus 0.2 μL screening solution; reservoir: 60 μL screening solution; 20°C). 1,200 conditions were screened. Under several conditions, *Mp*PR crystals appeared within 2 weeks. Conditions were optimized using the hanging-drop vapor-diffusion technique at 20°C. The optimized crystallization condition for *Mp*PR was as follows: 1 M ammonium sulfate, 0.1 M Bis-Tris (pH 5.5), 1% W/V PEG 3,350 at 20°C. Crystals were transferred to reservoir solution containing 20% (v/v) glycerol and flash-cooled with liquid nitrogen. Diffraction data were collected from cryo-cooled crystals at SSRF BL17U. Data were processed using HKL2000 (Otwinowski and Minor, 1997) and CCP4i (Winn et al., 2011). The resolution cut-off criteria were: (i) I/σ > = 2.0, (ii) CC_1__/__2_ (highest resolution shell) > 0.5.

The structure of *Mp*PR was solved by molecular replacement with MOLREP (Adams et al., 2010; Vagin and Teplyakov, 2010) using the structure of native raspberry ketone synthase from *Rubus idaeus* (PDB ID 6EOW) as a starting model. The molecular replacement solution was good, and an automatic model building was performed with Phenix (Adams et al., 2010). Additional model building was done manually with Coot (Emsley and Cowtan, 2004) and refined with Phenix. The final model of *Mp*PR was refined to 2.7 Å resolution. The final model for *Mp*PR was refined to R_work_ and R_free_ of 0.27/0.29 (Table 1).

**TABLE 1:** Structure data collection and refinement statistics.

**Protein**	***Mp*PR**
Data collection source	SSRL BL17U
PDB code	7EQL
**Data collection**	
Space group	P6_2_
Cell dimensions	
a, b, c (Å)	120.11,120.11,57.63
α, β, γ (°)	90.0, 90.0, 120.0
Resolution (Å)	104.02–2.72 (2.93–2.72)
Number of unique reflections	12,422
R_merge_	0.034 (0.473)
R_meas_	0.039 (0.539)
R_pim_	0.019 (0.255)
CC_1__/__2_	0.999 (0.873)
I/σI	19.6 (3.1)
Completeness (%)	96.30 (99.30)
**Refinement**	
Number of unique reflections	12,422
Number of test reflections	695
R_work_/R_free_	0.27/0.29 (0.39/0.41)
Number of atoms	
Protein	2,656
r.m.s.deviations	
Bond lengths (Å)	0.003
Bond angles (°)	0.583
MolProbity statistics	
Clashscore	9.89
Rotamer outliers (%)	2.20
Cβ outliers (%)	0
Ramachandran plot	
Favored (%)	97
Outliers (%)	0

*Highest resolution shell in parentheses.*

### Molecular Docking and Modeling Studies

All molecular docking studies were performed using Autodock4.2 package (Morris et al., 2009). Briefly, crystal structure of *Mp*PR enzyme was docked with (+)-pulegone. The molecule was added with non-polar hydrogens and assigned partial atomic charges using AutoDockTools (ADT) (Morris et al., 2009). The coordinates of NADP(H) and (+)-pulegone in *Mp*PR structure was generated based on the coordinates of hydroxybenzalacetone from the crystal structure of *Ri*DBR (PDB ID: 6EOW) and the coordinates of *p*-coumaryl aldehyde of the crystal of *At*DBR (PDB ID: 2J3J) in combination with CORINA Classic online service. A grid box with 40 × 40 × 40 grid points and 0.2 Å grid spacing centered roughly at the pulegone binding position was used as the searching space. 100 runs of Larmarckian Genetic Algorithm were performed to search the protein-ligand interactions. The results were clustered and ranked. Result analyses and figure rendering were performed using PyMOL. The structure of (−)-pulegone reductase from *N. tenuifolia* was modeled by the online artificial intelligence tfold2 program.^1^

### Data Availability

The crystal structure of *Mp*PR was deposited into Protein Data Bank under accession number 7EQL. UniProt IDs: Q6WAU0 for *Mp*PR; Q39172 for *At*DBR; Q9SLN8 for *Nt*DBR; G1FCG0 for *Ri*DBR; A0A5N5GUE7 for *Md*DBR. National Center Bioinfomratic Center (NCBI) accession number: MZ504956 for *Nt*PR; MZ504957 for *Ar*PR.

## Results

### Functional Characterization of Pulegone Reductases From *Mentha piperita* and *Nepeta tenuifolia* Revealed Substrate Selectivity Toward Enantiomers (+) and (−)-Pulegone

Pulegone reductase from *Mentha piperita* (*Mp*PR) that catalyzes the reduction of the C2–C8 double bond of (+)-pulegone to (−)-menthone using NADP(H) as a co-factor has been established for decades (Lange, 2015). Besides *M. piperita*, many other medicinal herbs, such as *Nepeta tenuifolia* and *Agastache rugosa* are also producers of essential oils, in which menthone and menthol are the major components (Chen et al., 2019). Intriguingly, it has been reported that *N. tenuifolia* and *A*. *rugosa* produce (+)-menthone and (+)-menthol as the major metabolites, in contrast to *M. piperita* that predominantly biosynthesizes (−)-menthone and (−)-menthol (Chen et al., 2019).

To investigate the underlying reasons for the substrate selectivity between *M. piperita* and *N. tenuifolia* or *A*. *rugosa*, we first compared the evolutionary relatedness of these three plants by performing molecular phylogenetic analysis based on a cascade of 28S-18S-5.8S rDNA sequences from their genomes. The results clearly showed that *M. piperita*, *N. tenuifolia*, *A. rugose*, *Sesamum indicum*, and *Amborella trichopoda* were clustered into a big group. More importantly, *M. piperita*, *N. tenuifolia*, and *A. rugose* were further classified into an individual subcluster, indicating that the three plants may be evolutionarily related (Supplementary Figure 2). Next, we analyzed menthone biosynthesis pathway in the genus *M. piperita* in which was well studied before. Limonene synthases, isopiperitenone reductase, and pulegone reductase have been proven to be able to catalyze the committed step in the biosynthesis of menthone until now (Supplementary Figure 1B; Ringer et al., 2003, 2005). Moreover, the structure-function relationships underlying the formation of limonene enantiomers in limonene synthases and candidate active site residues with critical roles in catalyzing reactions that involve accommodating reaction intermediates of opposite enantiomeric series have been identified (Hyatt et al., 2007; Srividya et al., 2020). However, there is no information concerning the biosynthesis mechanism of limonene downstream product enantiomers and relevant biosynthesis enzymes like isopiperitenone reductase or pulegone reductase so far. Therefore, we performed RNA-seq analysis on *N. tenuifolia* that produces (−)-pulegone and (+)-menthone in order to locate the candidate genes of (−)-pulegone reductase (Liu et al., 2021). Based on our *N. tenuifolia* RNA-seq data, three candidate genes of pulegone reductase (*cluster-16657.51589*, *cluster-16657.19187*, *cluster-16657.38628*) sharing high identities (∼65, ∼75, and ∼64%, respectively) with *Mp*PR were proposed to be *Nt*PR (Supplementary Figure 3). We then selected *cluster-16657.51589* as our target gene in view of its highest expression level in leaves and the leaves contain the highest ratio of monoterpenes (e.g., pulegone, menthone) among all the tissues of *N. tenuifolia* (Supplementary Figure 4). Subsequently, we performed sequence alignment analysis of *Nt*PR (*cluster-16657.51589*) and *Mp*PR together with another four DBRs (*At*DBR, *Nt*DBR, *Md*DBR, and *Ri*DBR) in the MDR superfamily. The alignment revealed that most residues in the proposed substrate binding pocket across all aligned MDR superfamily proteins are conserved (Supplementary Figure 5, labeled with green triangles). Moreover, *Nt*PR and *Mp*PR showed high sequence similarity with the major exception that six amino acid residues (Leu56, Tyr78, Phe281, Val282, Val284, and Tyr287 numbered as in *Mp*PR, Ser59, Asp81, Tyr283, Leu284, Tyr286, and Arg289 numbered as in *Nt*PR) in the proposed pulegone binding pocket are different (Supplementary Figure 5), suggesting these amino acids might contribute to substrate or product specificity. Thus, we cloned *Nt*PR and *Mp*PR genes and expressed the respective pulegone reductase in *Escherichia coli* BL21(DE3) strain for biochemical and structural studies (Supplementary Figure 6).

**FIGURE 1:**
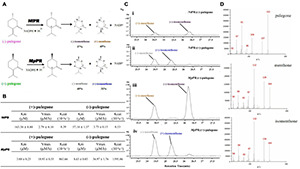
Functional characterization of (–)-pulegone reductase from *N. tenuifolia and* (+)-pulegone reductase from *M. piperita*. **(A)** Reduction of pulegone to menthone/isomenthone catalyzed by pulegone reductases from *N. tenuifolia* (*Nt*PR) and *M. piperita* (*Mp*PR). **(B)** Kinetic parameters of *Nt*PR or *Mp*PR that reduces (+)-pulegone or (–)-pulegone as the substrate, respectively. **(C,D)** *Nt*PR- and *Mp*PR-catalyzed *in vitro* conversion of pulegone to menthone/isomenthone monitored by chiral GC and GC/MS. Reactions were performed as described in Materials and Methods. Each reaction contained 20 μM substrate, and 30 μM *Mp*PR or 36 μM *Nt*PR. Left panel, GC chromatograms of methanolic extracts of the reactions: (i) (–)-pulegone + *Nt*PR + NADPH, (ii) (+)-pulegone + *Nt*PR + NADPH, (iii) (–)-pulegone + *Mp*PR + NADPH, and (iv) (+)-pulegone + *Mp*PR + NADPH. The horizontal axis represents retention time and the vertical axis represents relative abundance; right panel, GC/MS spectra of pulegone and menthone (SIM mode, *m/z*).

To experimentally validate the substrate specificity of *Nt*PR and *Mp*PR, we firstly carried out *in vitro* enzyme activity assays, where the pulegone reductase was fed with (+)- and (−)-pulegone, respectively. The products were analyzed and quantified using gas chromatography-mass spectrometry (GC-MS). The results clearly showed that *Nt*PR has a higher preference for (−)-pulegone to generate (−)-isomenthone (57%) and (+)-menthone (43%), confirmed by chiral GC and the fragments generated from electron impact mass spectrometry (EIMS) by comparison with standards (Figures 1A–D). In contrast, *Mp*PR is more inclined to convert (+)-pulegone to the major (−)-menthone (69%) and the minor (+)-isomenthone (31%) as reported previously (Figures 1A–D; Lange, 2015). Next, we plotted the Michaelis–Menten curve and calculated the enzyme kinetic parameters for *Nt*PR after feeding with (+)- or (−)-pulegones. Apparently, *Nt*PR has lower catalytic activities on (+)- or (−)-pulegone [K_cat_ 0.39 10^–4^s^–1^ for (+)-pulegone or 0.53 10^–4^s^–1^ for (−)-pulegone] compared to that of *Mp*PR [K_cat_ 863.66 10^–4^s^–1^for (+)-pulegone and 1595.06 10^–4^s^–1^ for (−)-pulegone]. *Nt*PR displayed higher binding affinity toward (−)-pulegone (K_m_ 57.18 μM) than that for (+)-pulegone (K_m_ = 163.30 μM), whereas the V_max_ for both (+)- and (−)-pulegones appears similar (Figure 1B), confirming that (−)-pulegone is the more favorable substrate that *Nt*PR can bind rather than (+)-pulegone. In comparison with *Nt*PR, *Mp*PR displayed higher binding affinity toward (+)-pulegone (K_m_ 3.00 μM) than that for (−)-pulegone (K_m_ 8.63 μM) (Figure 1B). To further investigate the substrate selectivity of *Mp*PR and *Nt*PR, we fed the pulegone reductases with other alkene double bond-containing substrates, including (+)-menthone, (−)-menthone, (+)-limonene, (−)-limonene, (+)-menthofuran, (−)-(1R,4S)-*p-*mentha-2,8-dien-1-ol, carveol, (−)-perilllc alcohol, and (−)-carvone. The results revealed that *Mp*PR and *Nt*PR can adopt (+)- and (−)-pulegones as substrate exclusively, and they do not show any activity toward other substrates (Supplementary Figure 7), suggesting *Mp*PR and *Nt*PR are the DBRs with high substrate specificity.

**FIGURE 2:**
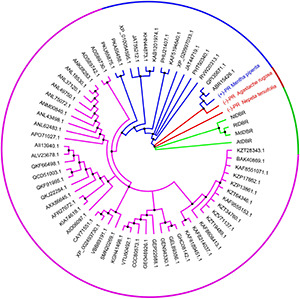
Phylogenetic analysis of alkene reductases from the MDR superfamily. The clades of alkene reductases from the MDR superfamily in which new identified pulegone reductase *N. tenuifolia* (–)-pulegone reductase (*Nt*PR), *A. rugose* (–)-pulegone reductase (*Ar*PR), and previous identified pulegone reductases including *M. piperita* (+)-pulegone reductase (*Mp*PR) located are highlighted in red and blue, respectively. The neighboring clade mainly bearing the double bond reductases, such as *Md*DBR, *At*DBR, *Nt*DBR, and *Ri*DBR from the MDR superfamily, the structures and functions of which have been previously investigated, is highlighted in green. The rest clade including other unrelated alkene reductases belonging to the MDR superfamily but the structures and functions of which have not been previously well studied is highlighted in purple. The alkene reductases IDs from the MDR superfamily mentioned above are referring to the National Center for Biotechnology Information (NCBI). All source species for the alkene reductases from the MDR superfamily used in the phylogenetic analysis are listed in Supplementary Table 3.

Using *Nt*PR sequence as a query, we further investigated the evolutionary relationship of *Nt*PR, *Mp*PR, and other DBRs in the MDR superfamily. 67 DBRs belonging to the MDR superfamily from plants that have complete genome data in KEGG database were chosen for the maximum-likelihood phylogenetic analysis. As expected, the phylogenetic tree revealed that *Nt*PR forms a unique clade, which suggests *Nt*PR may be evolutionarily distinct from other known MDR reductases (Figure 2 and Supplementary Figure 8). In contrast, the clade where *Mp*PR locates has more homologous members. The results inspired us to perform in-depth structural analysis of *Nt*PR and *Mp*PR toward the better understanding of substrate and product stereoselectivity.

### Structural Analyses of *Mentha piperita* and *Nepeta tenuifolia* Enzymes

**FIGURE 3:**
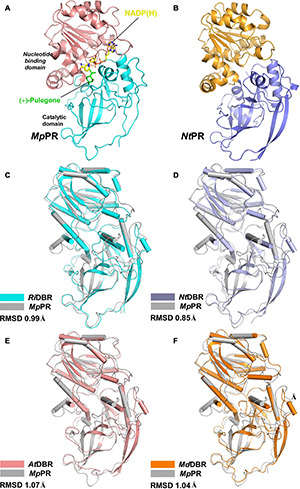
Overall structures of pulegone reductases from *Mentha piperita* and *Nepeta tenuifolia*. **(A)** Overall structure of (+)-pulegone reductase from *M. piperita* (*Mp*PR) bound with NADP(H). Residues 1–134 and 310–341 are colored cyan, and residues 135–309 are colored salmon. The NADP(H) and modeled (+)-pulegone are shown as yellow and green stick models, respectively. **(B)** Modeled structure of pulegone reductase from *N. tenuifolia* (*Nt*PR) using the tfold2 program. Residues 1–134 and 310–344 are colored blue, and residues 135–309 are colored orange. **(C)** Structure superimposition of *Mp*PR and *Ri*DBR, **(D)** structure superimposition of *Mp*PR and *Nt*DBR, **(E)** structure superimposition of *Mp*PR and *At*DBR. **(F)** Structure superimposition of *Mp*PR and *Md*DBR. the RMSDs of *Ri*DBR, *Nt*DBR, *At*DBR, and *Md*DBR with *Mp*PR are shown.

To elucidate the molecular basis underlying the differences in substrate and product specificities, we attempted to obtain co-crystals of *Mp*PR and *Nt*PR with NADP(H) and (±)-pulegone. Finally, the crystal structure of *Mp*PR was obtained at 2.7 Å resolution (Figure 3A), although the attempts to acquire crystal of *Nt*PR and co-crystals of the enzyme with either NADP^+^, (+)-pulegone or both NADP^+^ and (+)-pulegone failed, due to the hydrophobic and volatile nature of pulegone. Statistics of data collection and model refinement for *Mp*PR crystal are summarized in Table 1. Alternatively, the structure of *Nt*PR was *in silico* modeled using the tfold2 program (Figure 3B), an artificial intelligence-based online structural modeling tool. By structure superimposition analysis, the overall structures of both *Mp*PR and *Nt*PR are similar to the known MDR superfamily enzymes *Ri*DBR, *Nt*DBR, *At*DBR, and *Md*DBR (the RMSDs are 0.99, 0.85, 1.07, and 1.04 Å, Figures 3B–F), which form a different clade in the phylogenetic tree (in green, Figure 2).

The crystal structure of *Mp*PR revealed an asymmetric unit containing only one monomer of apo-*Mp*PR. The PDBePISA server calculations and gel filtration of the protein suggested that the biologically relevant form of the enzyme is a homodimer as reported previously for other MDR superfamily members (Supplementary Figure 9). The structure consists of two typical conserved N-terminal and C-terminal domains that are connected by a short loop. The N-terminal catalytic domain (residues 1–134 and 310–349) includes three a-helices and nine b-sheets forming a twisted partial b-barrel-like structure, while the C-terminal nucleotide coenzyme-binding Rossmann-fold domain (residues 135–309) features seven a-helices and six b-sheets, forming a typical six-stranded, parallel b-sheet sandwiched by three helices on each side (Figure 3A). The two domains are separated by a cleft containing a deep pocket that accommodates the cofactor and forms the active site (Figure 3A).

**FIGURE 4:**
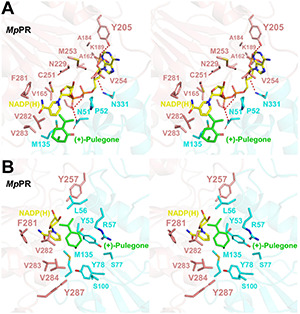
Structural modeling analysis of the NADP(H) and (+)-pulegone binding site of pulegone reductases from *Mentha piperita* (*Mp*PR). **(A)** Zoom-in stereo view shows the key residues of *Mp*PR involved in the interaction with NADP(H). Color codes are the same as those in Figure 3. Red dashed lines indicate hydrogen bonds. **(B)** Zoom-in stereo view shows the key residues of *Mp*PR involved in the interaction with (+)-pulegone. Color codes are the same as those in Figure 3.

We did not observe the unambiguous electronic density for NADP(H) in the map of co-crystal of *Mp*PR in complex with NADP(H), but two extra density peaks accounting for the phosphate group of NADP(H) were present, probably due to the crystal packing. Sequence alignment of *Mp*PR with other DBRs (e.g., *At*DBR and *Nt*DBR) in the MDR superfamily showed that most residues predicted to interact with NADP(H) are conserved and located at the nucleotide coenzyme-binding domain of *Mp*PR (Supplementary Figure 5, shown as purple rectangle). In order to locate the relative position of NADP(H) in *Mp*PR, we superimposed the phosphate group of NADP(H) in the crystal structure of *Mp*PR with that in *At*DBR (PDB ID 2J3J and 2J3K). The superimposed model exhibited that the residues Asn51, Ser164, Lys189, Tyr205, and Asn331 in *Mp*PR may form hydrogen bonds with the phosphate groups and the ribose rings of NADP(H) (Figure 4A). A number of hydrophobic residues consisting of Pro52, Tyr53, Met135, Ala162, Val165, Ala184, Cys251, Met253, Val254, Phe281, Val282, and Val283 were found surrounding the NADP(H) backbone to further stabilize the NADP(H) molecule (Figure 4A).

Due to the lack of co-crystal structure of *Mp*PR with the substrate, we docked (+)-pulegone into the *Mp*PR structure based on the ternary complex structure of its homolog *At*DBR (PDB ID 2J3J and 2J3K). In contrast to the predicted NADP(H) binding pocket, residues Tyr53, Leu65, Tyr78, Met135, Tyr257, Phe281, Val282, Val283, Val284, and Tyr287 form a hydrophobic network stabilizing (+)-pulegone mainly via hydrophobic and van der Waals interactions (Figure 4B). In addition, residues Ser77 and Ser100 also contribute to the binding of (+)-pulegone by van der Waals interaction. Residues Tyr53 and Arg57 may interact with the ketone moiety of (+)-pulegone through polar interactions to further stabilize the binding of (+)-pulegone (Figure 4B). All these residues form a feasible (+)-pulegone binding pocket in the structure of *Mp*PR.

### Validation of the Key Residues Involved in (+)-Pulegone Binding

**FIGURE 5:**
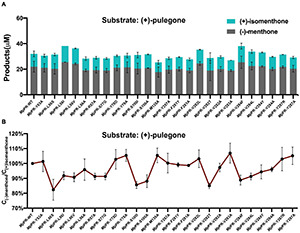
Site-directed mutagenesis analysis of the (+)-pulegone binding site of pulegone reductases from *Mentha piperita* (*Mp*PR). **(A)** Quantification of the reduction products (–)-menthone and (+)-isomenthone converted from the substrate (+)-pulegone by wild-type and mutated *Mp*PR. Reactions (0.4 mL) were performed in buffer (50 mM KH_2_PO_4_, 10% sorbitol, 1 mM DTT, pH 7.5) containing 20 μM substrate,10 mM NADPH tetrasodium salt hydrate, 6 mM glucose-6-phosphate, 20 U glucose-6-phosphate dehydrogenase and 30 μM *Mp*PR. 0.2 mL of n-hexane was added on the top of the reaction solution. Reaction was carried out at 31°C for 1 h. The concentration of products in n-hexane was determined by gas chromatography. Wild-type *Mp*PR and its mutants react under the same conditions. **(B)** The percentage ratio of the products (–)-menthone and (+)-isomenthone converted from **(A)**. Wild type’s ratio of (–)-menthone and (+)-isomenthone is normalized to 100%. Data are presented as mean ± S.D. of triplicate experiments.

To validate the predicted residues in pulegone binding pocket, we generated 25 single mutants by mutating 13 most relevant residues in the binding pockets of *Mp*PR and assessed the effect of point mutation on enzyme activity and the yield of final product (Supplementary Figure 6). *In vitro* enzyme catalysis assays showed that the wild-type *Mp*PR can convert (+)-pulegone to the products (−)-menthone and (+)-isomenthone with the ratio of 2:1 (Figure 5A and Supplementary Figure 10). The yield of the products decreased when the *Mp*PR mutants L56A, R57A, M135A, Y257A, F281A, V282A, V283A, V284A, and S77G were used in the reactions. Based on the docking model of *Mp*PR with NADP(H) and (+)-pulegone, the mutation R57A may disrupt the potential weak hydrogen bonding interaction with the ketone group of (+)-pulegone, thus reducing the enzyme activity slightly. The key residues Leu56, Val282, and Val284 in the binding pocket was proposed to interact with (+)-pulegone through hydrophobic effect. The hypothesis was evidenced by the mutation of Leu56, Val282, and Val284 to the non-polar residues with larger hydrophobic side chain (Leu56 to Ile and Val, Val282 to Leu, and Val284 to Phe, Leu, and Tyr) led to a remarkable increase in the yield of products when compared with the wild-type enzyme (Figure 5). Site-directed mutagenesis analysis validated the amino acid residues Leu56, Arg57, Ser77, Tyr257, Phe281, Val282, Val283, and Val284 in *Mp*PR binding pocket are critical to substrate binding and catalysis, consistent with the interactions revealed from the docking model of *Mp*PR in complex with NADP(H) and (+)-pulegone.

### Identification of *Mentha piperita* Residues Contributing to (+)- and (−)-Pulegone Specificity

**FIGURE 6:**
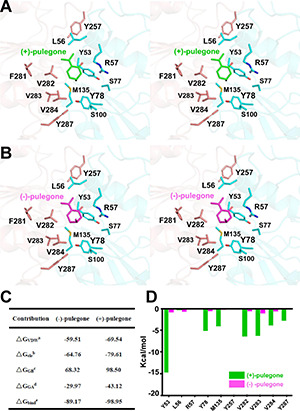
Structural docking results and predicted binding parameters of *Mentha piperita* (+)-pulegone reductase (*Mp*PR) bound with the substrate (+)-pulegone or (–)-pulegone by MD simulations. **(A)** Stereo view shows the key residues of *Mp*PR in the interaction with docked (+)-pulegone. **(B)** Stereo view shows the key residues of *Mp*PR in the interaction with docked (–)-pulegone. **(C)** Calculated intermolecular forces and binding free energies between *Mp*PR and (±)-pulegone by MD simulations. **ΔG_VDW_**^a^, Contribution to the free energy of binding from the van der Waals interaction; **ΔG_ele_**^b^, Contribution to the free energy of binding from the electrostatic interaction; **ΔG_GB_**^c^, Contribution to the free energy of binding from the polar interaction; **ΔG_GA_**^d^, Contribution to the free energy of binding from the non-polar interaction; **ΔG_bind_**^e^, Free energy of binding. **(D)** Strengths of hydrophobic interactions contributed by residues located at the potential pulegone binding pocket of *Mp*PR predicted by MD simulations.

Once the putative residues (i.e., Leu56, Arg57, Ser77, Tyr257, Phe281, Val282, Val283, and Val284) in the (+)-pulegone binding pocket of *Mp*PR were validated, we set out to explore the underlying mechanism on why *Mp*PR has preference to (+)-pulegone rather than (−)-pulegone as observed in our GC-MS analysis and enzyme kinetic study (Figure 1). Firstly, (+)- and (−)-pulegones were docked into the crystal structure of *Mp*PR with NADP(H), respectively, and we observed that the angle of C-5 methyl group (5-Me) of pulegone in the binding pocket likely determines the substrate selectivity of *Mp*PR. Specifically, 5-Me of (+)-pulegone perfectly interacts with the hydrophobic network constituted by Tyr53, Leu56, Tyr78, M135, Val282, Val283, Val284, and Tyr287, stabilizing the binding between *Mp*PR and (+)-pulegone. In contrast, 5-Me of (−)-pulegone does not totally fit into the hydrophobic network and may destabilize the binding with *Mp*PR (Figures 6A,B). To validate the hypothesis, we calculated the forces and energies generated during the binding between pulegone and *Mp*PR using molecular dynamic (MD) simulations. The free energy of binding (ΔG_bind_^e^) calculated for (+)-pulegone to *Mp*PR (–98.95 kcal/mol) is significantly lower than that of (−)-pulegone (–89.17 kcal/mol), indicating a tendency to adopt (+)-pulegone as the substrate by *Mp*PR (Figure 6C). Furthermore, the non-polar interaction (ΔG_GA_^d^) might contribute to the free energy of binding to the most extent with the values of –29.97 kcal/mol for (+)-pulegone and –43.12 kcal/mol for (−)-pulegone (Figure 6C). The MD simulations also predicted and compared the contribution of each amino acid residue involved in the non-polar interaction with (+)- and (−)-pulegones. MD simulations results showed that the residues Tyr78, Met135, Val282, Val283, Val284, Tyr287, and particularly Tyr53 constitute much stronger hydrophobic interactions with (+)-pulegone compared with those for (−)-pulegone, suggesting these residues are highly likely involved in stereoselectivity of the substrate (Figure 6D). The calculated results are highly consistent with our hypothesis that the interaction between these residues and 5-Me of (+)-pulegone stabilize their binding and thus led to the substrate specificity.

**FIGURE 7:**
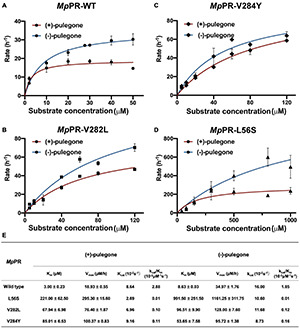
Measurement of kinetic parameters for the wild-type *Mentha piperita* (+)-pulegone reductase (*Mp*PR) and its mutants. **(A–D)** Michaelis Menten plot of for wild-type, V282L, V284Y, and L56S *Mp*PR using (+)-pulegone or (–)-pulegone as the substrate. Reactions were performed as described in Materials and Methods. Supplementary Table 1 provides enzyme concentration and substrate concentration for each reaction. **(E)** Summary of enzyme kinetic parameters of the wild-type *Mp*PR and its mutants. Values are means ± S.D. and error bars indicate the S.D. for three biological replicates.

To further experimentally investigate the importance of these predicted residues involved in substrate stereoselectivity, we mutated the hydrophobic residues Leu56, Val282, and Val284 in the binding pocket of (+)-pulegone in *Mp*PR to the corresponding residues Ser59, Leu285, and Tyr287 in *Nt*PR. The enzyme kinetic measurements of the wild-type *Mp*PR revealed that its binding affinity to (+)-pulegone (K_m_ ∼3.00 μM) is about threefold higher than that to (−)-pulegone (K_m_ ∼8.63 μM). The mutations L56S, V282L, and V284Y led to 8–100-fold decrease in the binding affinity to both (+)- and (−)-pulegone (Figures 7A–E). Importantly, the mutation V282L and V284Y almost diminished the substrate stereoselectivity of *Mp*PR as evidenced by the very similar K_m_ values for both (+)- and (−)-pulegone, possibly attributed to the larger side chains of the mutants that strengthen the hydrophobic interaction with (−)-pulegone (Figures 7A–E). In contrast, the mutation L56S did not display a significant effect on the substrate selectivity, suggesting Leu56 is not involved in the substrate specificity.

### Identification of *Mentha piperita* Residues Contributing to the Stereoselectivity of the Products (−)/(+)-Menthone and (+)/(−)-Isomenthone

**FIGURE 8:**
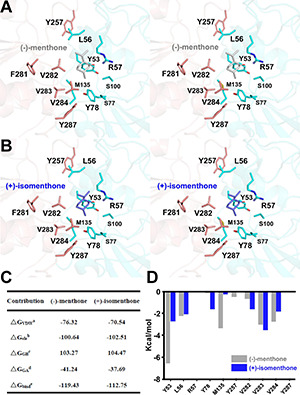
Structural docking results and predicted binding parameters of *Mentha piperita* (+)-pulegone reductase (*Mp*PR) with the products (–)-menthone or (+)-isomenthone by MD simulations. **(A)** Stereo view shows the key residues of *Mp*PR interacting with the product (–)-menthone. **(B)** Stereo view shows the key residues of *Mp*PR interacting with the product (+)-isomenthone. **(C)** Calculated intermolecular forces and free binding energies between *Mp*PR and (–)-menthone or (+)-isomenthone by MD simulations. The parameters are the same as in Figure 5C. **(D)** Strengths of hydrophobic interactions contributed by the binding pocket residues of *Mp*PR predicted by MD simulations.

*In vitro* enzyme catalysis assays coupled with GC-MS analysis showed that the wild-type *Mp*PR can convert (+)-pulegone to the products (−)-menthone and (+)-isomenthone with the ratio of 2:1. This observation encouraged us to explore the potential mechanism on the product stereoselectivity of *Mp*PR. Given that the only difference between the structures of (−)-menthone and (+)-isomenthone lies in the angle of the 2-isopropyl group (2-iPr), we therefore proposed that the product stereoselectivity may be attributed to the stereochemistry of 2-iPr, which could result in different interaction strengths and binding affinities to *Mp*PR. To test this hypothesis, we first docked (−)-menthone and (+)-isomenthone into the crystal structure of *Mp*PR, respectively. The docking model showed that the 2-iPr of (−)-menthone is well positioned in the binding pocket of *Mp*PR and stabilized by Tyr53, Leu56, Val282, Val283, and Val284, while the 2-iPr of (+)-isomenthone crashes the main chain of residue Tyr53 in the pocket, thus requiring higher energies to generate and stabilize with *Mp*PR (Figures 8A,B). Subsequently, the binding affinities of (−)-menthone and (+)-isomenthone to *Mp*PR were calculated using MD simulations. The enzyme kinetic parameters supported our deduction from the structural analysis as the free binding energy (ΔG_bind_^e^) for (−)-menthone (–119.43 kcal/mol) is higher than that for (+)-isomenthone (–112.75 kcal/mol) (Figure 8C). Moreover, the MD simulations also predicted the potential contributions of the surrounding residues to the non-polar hydrophobic interactions with (−)-menthone or (+)-isomenthone. The residues Tyr53, Leu56, Tyr78, Met135, Val282, Val283, and Val284 have major contributions to the binding of the products (Figure 8D). Particularly, Tyr53, M135, and V284 are inclined to stabilize (−)-menthone rather than (+)-isomenthone as revealed by the lower ΔG_GA_^d^ values, whereas Tyr78, Val282, and Val283 have larger contribution to the stabilization of (+)-isomenthone through hydrophobic interactions (Figure 8D).

Next, we performed an in-depth analysis of the site-directed mutagenesis data to evaluate the impact of these residues on the stereoselectivity of products (−)-menthone and (+)-isomenthone. The ratio of the products (−)-menthone and (+)-isomenthone was calculated for each mutant and normalized based on those for the wild-type *Mp*PR. The results revealed that the mutants L56S, L56I, L56V, S100I, S100A, V282T, V284F, V284L, V284Y, and V284A significantly reduced the ratio of (−)-menthone to (+)-isomenthone, but have little impact on the total yield of the products (Figures 5A,B), suggesting that the residues Leu56, Ser100, Val282, and Val284 may play critical roles in the stereoselectivity of the products (−)-menthone and (+)-isomenthone, which was consistent with our docking and MD simulation results described above.

In the enzyme catalysis assay and GC-MS analysis, we found that *Mp*PR can also adopt (−)-pulegone as its substrate but with low affinity to generate (+)-menthone (∼46%) and the (−)-isomenthone (∼54%) (Figure 1C, Supplementary Figure 10, and Supplementary Table 1). To investigate the stereoselectivity of *Mp*PR for products (+)-menthone and (−)-isomenthone, we performed docking study and MD simulations. The docking analysis show that the product either (+)-menthone or (−)-isomenthone can well fit the proposed product binding pocket and does not have big crash with the surrounding residues (Supplementary Figures 11A,B), indicating (+)-menthone or (−)-isomenthone may be all favored by *Mp*PR. The MD simulations results supported the hypothesis because the free binding energy of product (+)-menthone (–141.26 kcal/mol) and (−)-isomenthone (–116.74 kcal/mol) is both relatively low (Supplementary Figure 11C), which is consistent with our GC-MS data. Furthermore, the residues Y53, L56, V282, and V284 in the binding pocket of *Mp*PR are predicted to mainly contribute to the non-polar hydrophobic interactions with (+)-menthone and (−)-isomenthone in the MD simulations (Supplementary Figure 11D). To experimentally validate these residues responsible for product stabilization, we performed mutagenesis analysis and assayed the abilities of mutants to convert (−)-pulegone to (+)-menthone and (−)-isomenthone. Compared with the wild-type enzyme, the mutation L56S, Y78D, and V282L decreased the ratio of products (+)-menthone and (−)-isomenthone, like the results obtained using the native substrate (+)-pulegone (Supplementary Figure 12). In contrast, the mutation L56I, L56V, R57A, Y78A, M135A, V282A, and V284A significantly improved the percentage yield of (+)-menthone, suggesting the residues Leu56, Arg57, Tyr78, Met135, Val282, and V284 might be critical to the stereoselectivity of products (+)-menthone and (−)-isomenthone (Supplementary Figure 12B).

## Discussion

Monoterpenes are constituents of essential oils and oleoresins used commercially in the flavor, fragrance, and medicine industries. Currently, monoterpenes are mainly extracted from plants using chemical methods, which is costly and environmentally unfriendly. Moreover, the yield of monoterpenes isolated from natural or engineered plants remain unsatisfactory and do not meet the large need in industry. Importantly, most of the medicinal plants grow slowly, and the planting area is limited due to the climate and other environmental conditions (Wang et al., 2015). Although chemical synthesis is another option, multiple chiral centers in most monoterpene structures pose a big challenge to total chemical synthesis and the cost to obtain enantiomerically pure monoterpenes is high. Thus, looking for alternative sustainable supply of industrially important monoterpenes is of great importance. The development of synthetic biology and metabolic engineering tools provides great opportunities for heterologous biosynthesis of monoterpenoids in microbial cells (Oswald et al., 2007; Herrero et al., 2008; Fischer et al., 2011; Lange, 2015; Zebec et al., 2016; Rajput et al., 2018; Bergman et al., 2019; Gao et al., 2020). Industrial-scale production of certain terpenoids using engineered microorganisms have been achieved, such as artemisinin acid and ginsenosides (Paddon et al., 2013; Wang et al., 2015; Wei et al., 2015; Talman et al., 2019). To achieve this goal, elucidation of monoterpenoid biosynthetic pathways, especially the key biosynthetic enzymes, is extremely essential the reconstruction of monoterpenoid pathways in microbial chassis cells. Considerable attention has been paid on the mechanism of the cyclization reactions in monoterpenoid biosynthesis, however, many fascinating puzzles and stereochemical anomalies remain unclear (Finefield et al., 2012).

(−)-Menthone is the second most abundant monoterpene [15–20% (*v/v*)] of peppermint essential oil and the substrate of (−)-menthol, which also represents anti-inflammatory, antiviral, and anti-bacterial activity (Shalayel et al., 2016). Natural (−)-menthone is crystallized from dementholized peppermint or cornmint essential oil, the source of which is limited to plants. Commercial (−)-menthone is always sold as a mixture with up to 29% (*v/v*) (+)-isomenthone (Lange, 2015). (+)-Menthone is the opposite chirality compound, occupying 20–30% (*v/v*) in *N. tenuifolia* essential oil, and display similar activities as (−)-menthone (Chen et al., 2017; Liu et al., 2018). The major source of commercial (+)-menthone is medicinal plants (Marumoto et al., 2017), and the yield is often varied due to the source and status of the plants. Therefore, achieving heterologous production of menthone in microbial host is a promising way for reproducible and high-titer production of enantiomerically pure menthone in industry.

Pulegone reductases catalyze the formation of menthone and determine the substrate specificity and product stereoselectivity. However, the structure of pulegone reductase and the underlying mechanism for substrate specificity and product stereoselectivity remain largely unknown. The implementation of a synthetic biology approach to the production of menthone was also hampered by the absence of the mechanism studies for pulegone reductase. In our study, we characterized a novel (−)-pulegone reductase from *Nepeta tenuifolia* (*Nt*PR), which displays opposite substrate specificity and product stereoselectivity to those for (+)-pulegone reductase isolated from *Mentha piperita* (*Mp*PR). Comparative analysis of the structures, key amino acids residues in the pulegone binding pocket, and enzyme kinetics for *Nt*PR and *Mp*PR using the combined bioinformatics, structure biology and biochemistry approaches led us to better understand the underlying mechanism of the substrate and product stereoselectivity. Through site-directed mutagenesis, we obtained several *Mp*PR mutants that improve the percentage of (−)-menthone to 70% and of (+)-menthone to 68%. The recombinant pulegone reductase could be further engineered to increase the production and percentage of menthone. Therefore, increasing the ratio/yield of major product menthone in the enzymatic reaction is critical to heterologous biosynthesis of menthone in microbial hosts.

Recently, molecular reaction mechanisms have been proposed for several DBRs, such as *At*DBR (Youn et al., 2006) and *Ri*DBR, in which the conjugated double bond of the substrate is in equilibrium with an α,β-conjugated enolate intermediate. In this condition, a hydride transfer occurs from the C-4 of the nicotine amide of NADP(H) (catalytic carbon) to the β carbon of the enolate intermediate, with a subsequent protonation of its α carbon. In *At*DBR, this process is facilitated by the stabilization of the propenal transition state by a π-π interaction between Tyr53 and the phenolic ring of the substrate *p*-coumaryl aldehyde (Youn et al., 2006). In *Ri*DBR ternary structure, a π-π stacking between the substrate hydroxybenzalacetone and nicotinamide aromatic rings is observed, with a hydride transfer distance of 3.06 Å to the alkene double bond (Simon et al., 2017). For *Mp*PR and *Nt*PR (Mansell et al., 2013), a π-π stacking may be formed between the double bond from the substrate pulegone and nicotinamide aromatic rings to facilitate hydride transfer (Figure 4).

Our extensive structural and biochemical analyses on *Mp*PR and *Nt*PR in this study provide a novel insight into the solution to meet the growing demand for high-quality natural (±)-menthone products in the future. Recently, we discovered another novel pulegone reductase from *Agastache rugosa* (*Ar*PR) through genomic analysis of *A. rugosa*, a medicinal plant belonging to the genus of *Labiatae* that produces therapeutic volatile oils. *Ar*PR is evolutionarily related to *Nt*PR and showed a higher preference for (−)-pulegone over (+)-pulegone (Supplementary Figure 13), suggesting (−)-pulegone reductase may be widely spread in medicinal plants of genera *Nepeta* and *Labiatae*. Pulegone reductase discovered in our study provides a valuable reservoir of genetic resources for biocatalytic applications, and an example to explore the stereoselectivity of monoterpenoid biosynthetic enzymes in plants. The crystal structures of *Mp*PR, *Nt*PR, *Pp*PR, or *Ar*PR in complex with its substrate and products are anticipated in the future for the comprehensive understanding of the stereoselectivity of pulegone reductases.

## Data Availability Statement

The datasets presented in this study can be found in online repositories. The names of the repository/repositories and accession number(s) can be found below: http://www.wwpdb.org/, 7EQL.

## Author Contributions

CL, QW, IL, NS, BL, and WL designed experiments. QG and CL performed the bulk of the experiments. QG, CL, IL, and NS contributed to gene identification and cloning, protein expression, purification, and crystallization. JL, LL, QG, NS, and CL contributed to enzymatic assay experiments and oil distillation. SZ contributed to molecular phylogenetic analysis. JL contributed to molecular dynamic simulations. CL, ZS, QW, and WL analyzed the data and wrote the manuscript. QW, BL, and WL conceived the project. All authors contributed to the article and approved the submitted version.

## Conflict of Interest

The authors declare that the research was conducted in the absence of any commercial or financial relationships that could be construed as a potential conflict of interest.

## Publisher’s Note

All claims expressed in this article are solely those of the authors and do not necessarily represent those of their affiliated organizations, or those of the publisher, the editors and the reviewers. Any product that may be evaluated in this article, or claim that may be made by its manufacturer, is not guaranteed or endorsed by the publisher.

## Footnotes

1
https://drug.ai.tencent.com/en
