# Corrigendum: Functional Characterization and Structural Insights Into Stereoselectivity of Pulegone Reductase in Menthol Biosynthesis

**DOI:** 10.3389/fpls.2021.828351

**Published:** 2022-01-14

**Authors:** Chanchan Liu, Qiyu Gao, Zhuo Shang, Jian Liu, Siwei Zhou, Jingjie Dang, Licheng Liu, Iris Lange, Narayanan Srividya, B. Markus Lange, Qinan Wu, Wei Lin

**Affiliations:** ^1^School of Pharmacy, Nanjing University of Chinese Medicine, Nanjing, China; ^2^Jiangsu Collaborative Innovation Center of Chinese Medicinal Resources Industrialization, Nanjing, China; ^3^Department of Pathogen Biology, School of Medicine and Holistic Integrative Medicine, Nanjing University of Chinese Medicine, Nanjing, China; ^4^CAS Key Laboratory of Quantitative Engineering Biology, Guangdong Provincial Key Laboratory of Synthetic Genomics and Shenzhen Key Laboratory of Synthetic Genomics, Shenzhen Institute of Synthetic Biology, Shenzhen Institutes of Advanced Technology, Chinese Academy of Sciences, Beijing, China; ^5^Institute of Biological Chemistry and M.J. Murdock Metabolomics Laboratory, Washington State University, Pullman, WA, United States; ^6^State Key Laboratory of Natural Medicines, China Pharmaceutical University, Nanjing, China

**Keywords:** pulegone reductase, stereoselectivity, molecular dynamics simulations, menthol biosynthesis, *Mentha piperita*

In the published article, there was an error regarding the affiliations for Chanchan Liu. As well as having affiliation 1, they should also have affiliation 2 “Jiangsu Collaborative Innovation Center of Chinese Medicinal Resources Industrialization, Nanjing, China.”

The authors apologize for this error and state that this does not change the scientific conclusions of the article in any way. The original article has been updated.

## Publisher's Note

All claims expressed in this article are solely those of the authors and do not necessarily represent those of their affiliated organizations, or those of the publisher, the editors and the reviewers. Any product that may be evaluated in this article, or claim that may be made by its manufacturer, is not guaranteed or endorsed by the publisher.

