# Supplementary material for: Functional Characterization and Structural Insights Into Stereoselectivity of Pulegone Reductase in Menthol Biosynthesis
Source: Front Plant Sci. 2021 Nov 30;12:780970. doi: 10.3389/fpls.2021.780970 (PMC8670242; doi:10.3389/fpls.2021.780970)
Supplement: Supplementary file 1 [file Presentation_1.pdf]

Supplementary Materials

Functional characterization and structural insights into stereoselectivity of pulegone reductase in menthol biosynthesis

Chanchan Liu [1,#], **Qiyu Gao** [2,#], **Zhuo Shang** [2], **Jian Liu** [1], **Siwei Zhou** [3], **Jingjie Dang** [1], **Licheng Liu** [1], **Iris Lange** [4], **Narayanan Srividya** [4], **B. Markus Lange** [4], **Qinan Wu** [1,5,*], **Wei Lin** [1,2,5,6,*]

[1] School of Pharmacy, Nanjing University of Chinese Medicine, Nanjing, China.

[2] Department of Pathogen Biology, School of Medicine & Holistic Integrative Medicine, Nanjing University of Chinese Medicine, Nanjing, China.

[3] CAS Key Laboratory of Quantitative Engineering Biology, Guangdong Provincial Key Laboratory of Synthetic Genomics and Shenzhen Key Laboratory of Synthetic Genomics, Shenzhen Institute of Synthetic Biology, Shenzhen Institutes of Advanced Technology, Chinese Academy of Sciences, Shenzhen, 518055, China.

[4] Institute of Biological Chemistry and M.J. Murdock Metabolomics Laboratory, Washington State University, Pullman, WA 99164-7411, USA

[5] Jiangsu Collaborative Innovation Center of Chinese Medicinal Resources Industrialization, Nanjing, China

[6] State Key Laboratory of Natural Medicines, China Pharmaceutical University, Nanjing, China

*****, Corresponding Authors
E-mail addresses: **Qinan Wu**: wuqn@njucm.edu.cn OR **Wei Lin**: weilin@njucm.edu.cn

[#], These authors contributed equally to this work.

SUPPLEMENTARY MATERIALS AND METHODS

Molecular Phylogenetic Analysis of *M. piperita, N. tenuifolia* and *A. rugosa* using Maximum Likelihood Method.

The phylogenetic tree was constructed based on a cascade of 28S-18S-5.8S rDNA sequences from the genomes of *M. piperita, N. tenuifolia, A. rugosa, Arabidopsis thaliana, Glycine max, Amborella trichopoda, Sesamum indicum, Vitis vinifera*, and *Oryza sativ*a. The evolutionary history was inferred by using the Maximum Likelihood method based on the Tamura-Nei model (Tamura and Nei, 1993). The bootstrap consensus tree inferred from 1000 replicates is taken to represent the evolutionary history of the taxa analyzed (Tamura et al., 2007;Tamura et al., 2013;Kumar et al., 2018). Branches corresponding to partitions reproduced in less than 50 % bootstrap replicates are collapsed. The percentage of replicate trees in which the associated taxa clustered together in the bootstrap test (1000 replicates) are shown next to the branches (Tamura et al., 2007;Tamura et al., 2013;Kumar et al., 2018). Initial tree(s) for the heuristic search were obtained automatically as follows. When the number of common sites is < 100 or less than one fourth of the total number of sites, the maximum parsimony method was used; otherwise BIONJ method with MCL distance matrix was used. The tree is drawn to scale, with branch lengths measured in the number of substitutions per site. The null hypothesis of equal evolutionary rate throughout the tree was rejected at a 5 % significance level ($p < 0.05$). Evolutionary analyses were conducted in MEGA11 software (Tamura et al., 2013;Kumar et al., 2018).

Quantitative Real-Time Polymerase Chain Reaction (qRT-PCR).

qRT-PCR was conducted to confirm the expression of the gene *pulegone reducase* from *N. tenuifolia*, the ribosome gene *EF-1α was* used as internal reference gene. We performed the assays at least three independent biological replicates and three technical replicates. RNA yield and quality were assessed by agarose gel electrophoresis. RNAprep Pure Plant Plus Kit (Tiangen Biotech) was used with 0.5 μg of total RNA to synthesize the first-strand cDNA. The qRT-PCR reactions were carried out using the QuantStudio 3 Real-Time PCR Systems (Applied Biosystems). ChamQ Universal SYBR qPCR (Vazyme Biotech) was used to prepare qRT-PCR reactions with 1 μL of diluted cDNA as a template. The primers used in this assay were shown in **Table S2**. The reaction systems and steps were performed according to the manufacturer’s instructions. Relative gene expression levels were calculated according to the 2-ΔΔCT method.

Simultaneous Distillation-Extraction of Essential Oils.

Root, stem, and leaf of *N. tenuifolia* were harvested when plants were at the full-flower stage (100 days after germination). 10 g of fresh sample material and several zeolites were transferred to a 150 mL round bottom flask with moderate deionized water for subsequent distillation using a modified Likens-Nickerson apparatus and n-hexane as the carrier solvent. An aliquot of the 10 mL n-hexane fraction, which contained the volatile oil constituents, was transferred to a brown glass bottle for further analysis.

Phylogenetic Analysis of Double Bond Reductases (DBR) in MDR superfamily.

67 protein sequences from the MDR superfamily enzymes including *Mp*PR, *At*DBR, *Md*DBR, *Ri*DBR were gathered by using *Mp*PR (UniProt ID: Q6WAU0), *At*DBR (UniProt ID: Q39172), *Md*DBR

(UniProt ID: A0A5N5GUE7) and *Ri*DBR (UniProt ID: G1FCG0) as searching template for Blast e-value cut-off at 1×10^{-25}. All the sequences were aligned using CLUSTAL W and analyzed using MEGA 11.0 (Tamura et al., 2013;Kumar et al., 2018), A Neighbor-Joining tree was constructed via the bootstrap method with 1000 replications (Tamura et al., 2013;Kumar et al., 2018)). The final map was drawn using the FigTree software.

Molecular Dynamics Simulations.

The starting structure of *Mp*PR monomer was extracted from the crystal structure of *Mp*PR. Two substrates (+)-pulegone and (-)-pulegone, and four products (-)-menthone, (+)-isomenthone, (+)-menthone, and (-)-isomenthone were analyzed as the potential ligands. A 100 ns long molecular dynamics simulation was performed with the AMBER16 software package. The force field parameters for the protein and ligands were generated with Amber ff14SB and general Amber force field, respectively. The model was solvated in a truncated octahedron box of the transferable interaction potential (TIP3P) water molecules with a margin distance of 10 Å. Sodium ions were added to neutralize the systems. The particle mesh Ewald (PME) method was used for long-range electrostatic interactions with a cutoff distance of 10 Å. Next, energy minimization was performed on the system through 2500 steps of steepest descent, followed by 2500 steps of conjugate gradients. After energy minimization, the whole system was gradually heated from 0 to 300 K for 60 ps under NVT, followed by a 600 ps NPT simulation at 1 atm, with harmonic restraints of 2 kcal/(mol*Å2) on the complex. Finally, the 100 ns of MD production was performed at 300 K with 1.0 atm pressure. The temperature and pressure were kept constant using a Langevin thermostat and a Langevin barostat, respectively. All hydrogen atoms were constrained by the SHAKE algorithm, and the time step was 2 fs. The resulting trajectories were analyzed with the AMBER16 module CPPTRAJ. At last, the binding-free energies (ΔG_{bind}) of the complexes were calculated by using MM-GBSA programs.

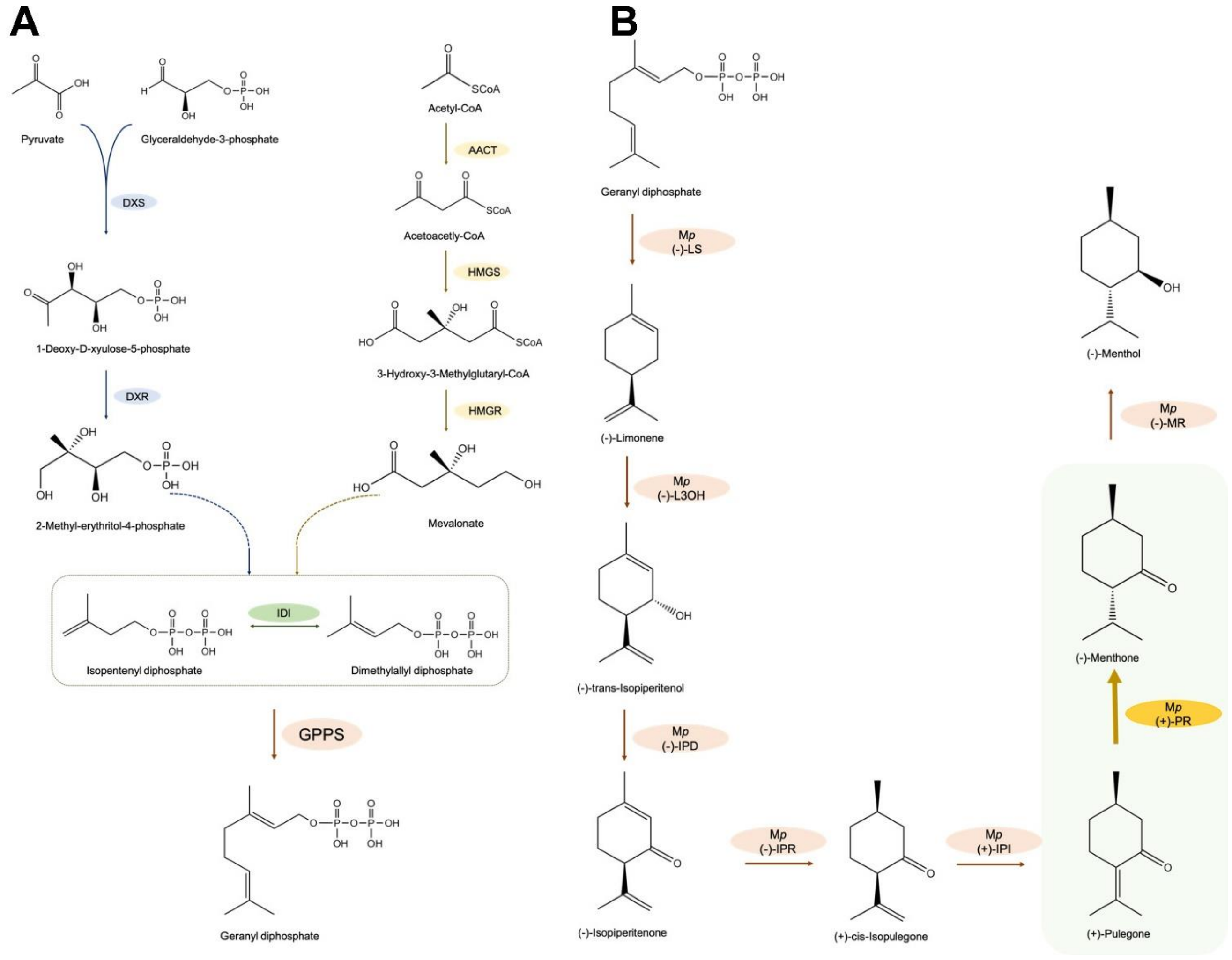

Supplementary Figure 1. Complete menthol biosynthetic pathway. (A)-(B) Upstream and downstream menthone biosynthetic pathway. DXS, 1-deoxy-D-xylulose 5-phosphate synthase; DXR, 1-deoxy-D-xylulose 5-phosphate reductoisomerase; ACCT, acetoacetyl coenzyme A thoilase; HMGS, 3-hydroxy-3-methyl glutaryl coenzyme A synthase; HMGR, 3-hydroxy-3-methyl glutaryl coenzyme A reductase; IDI, Isoprene pyrophosphate isomerase; GPPS, geranyl diphosphate synthase; LS, limonene synthase; L3OH, limonene-3-hydroxylase; IPD, trans-isopiperitenol dehydrogenase; IPR, isopiperitenone reductase; IPI, *cis*-isopulegone isomerase; PR, pulegone reductase; MR, menthone reductase.

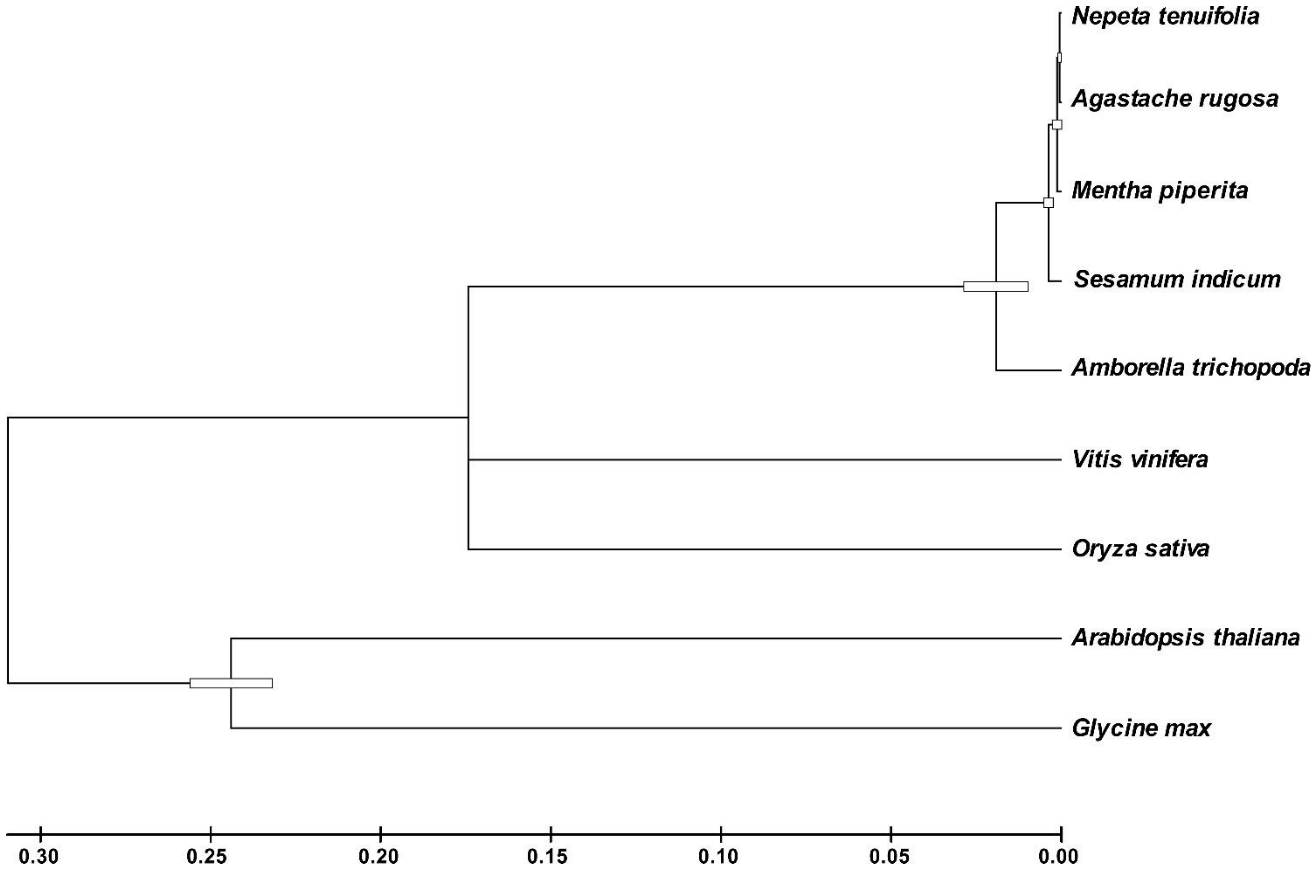

Supplementary Figure 2. Molecular phylogenetic analysis of *M. piperita*, *N. tenuifolia* and *A. rugosa* by Maximum Likelihood method. In order to analyze the potential evolutionary relatedness of the three plant species *Mentha piperita*, *Nepeta tenuifolia* and *Agastache rugosa*, the phylogenetic tree was constructed based on a cascade of 28S-18S-5.8S rDNA sequences from their genomes, using *Arabidopsis thaliana*, *Glycine max*, *Amborella trichopoda*, *Sesamum indicum*, *Vitis vinifera*, and *Oryza sativa* as the outgroup members. The scale bar indicates 0.05 amino acid substitutions per amino acid position. Please see *Materials and Methods* for more detail.

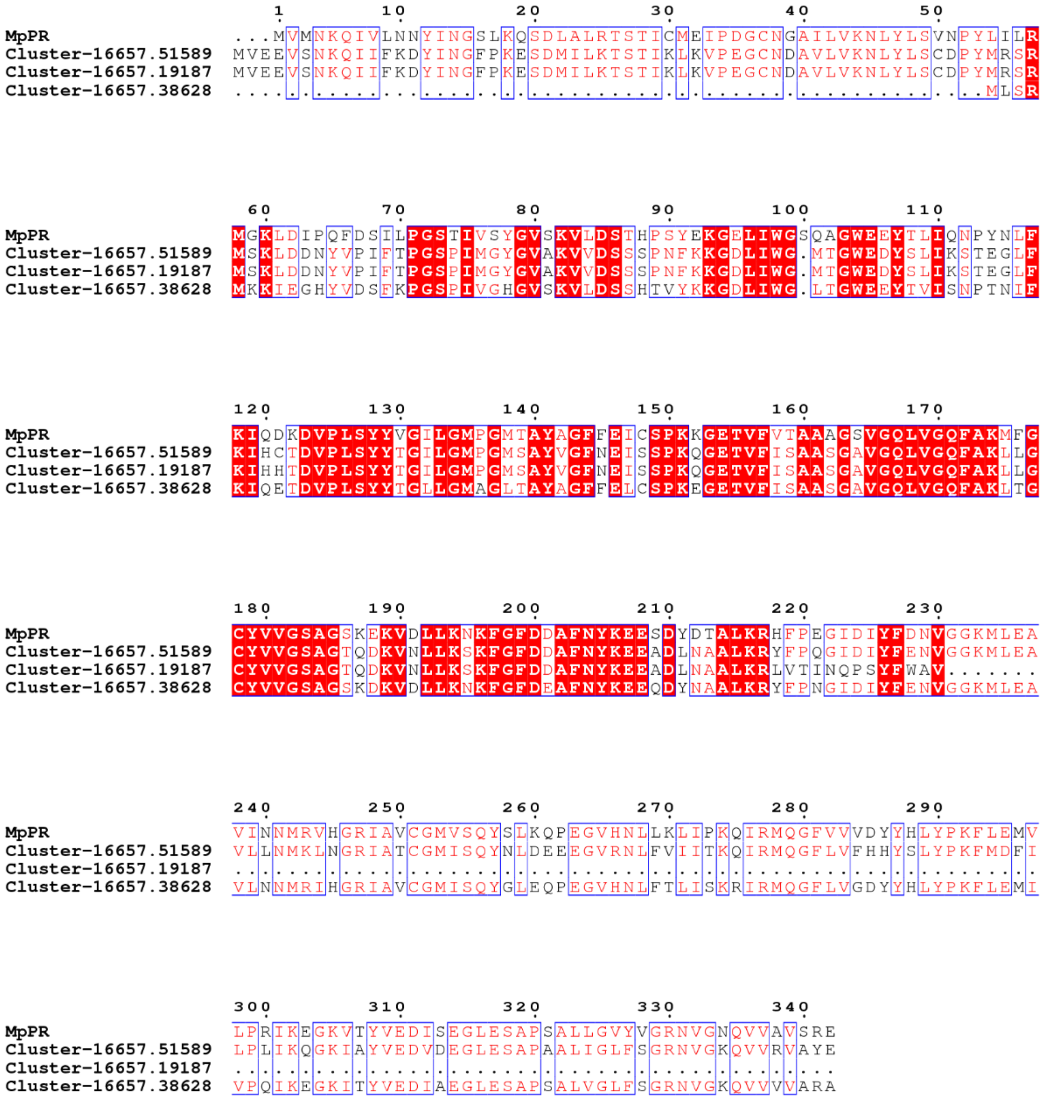

Supplementary Figure 3. Sequence alignment of *Mp*PR and predicted PRs from *Nepeta tenuifolia*. The invariant residues are highlighted in red, and conserved amino acids are boxed. *Mp*PR, pulegone reductase from *Mentha piperita*; Cluster-16657.51589, Cluster-16657.19187 and Cluster-16657.38628 are predicted pulegone reductase from *Nepeta tenuifolia*. The residue numbering is according to *Mp*PR.

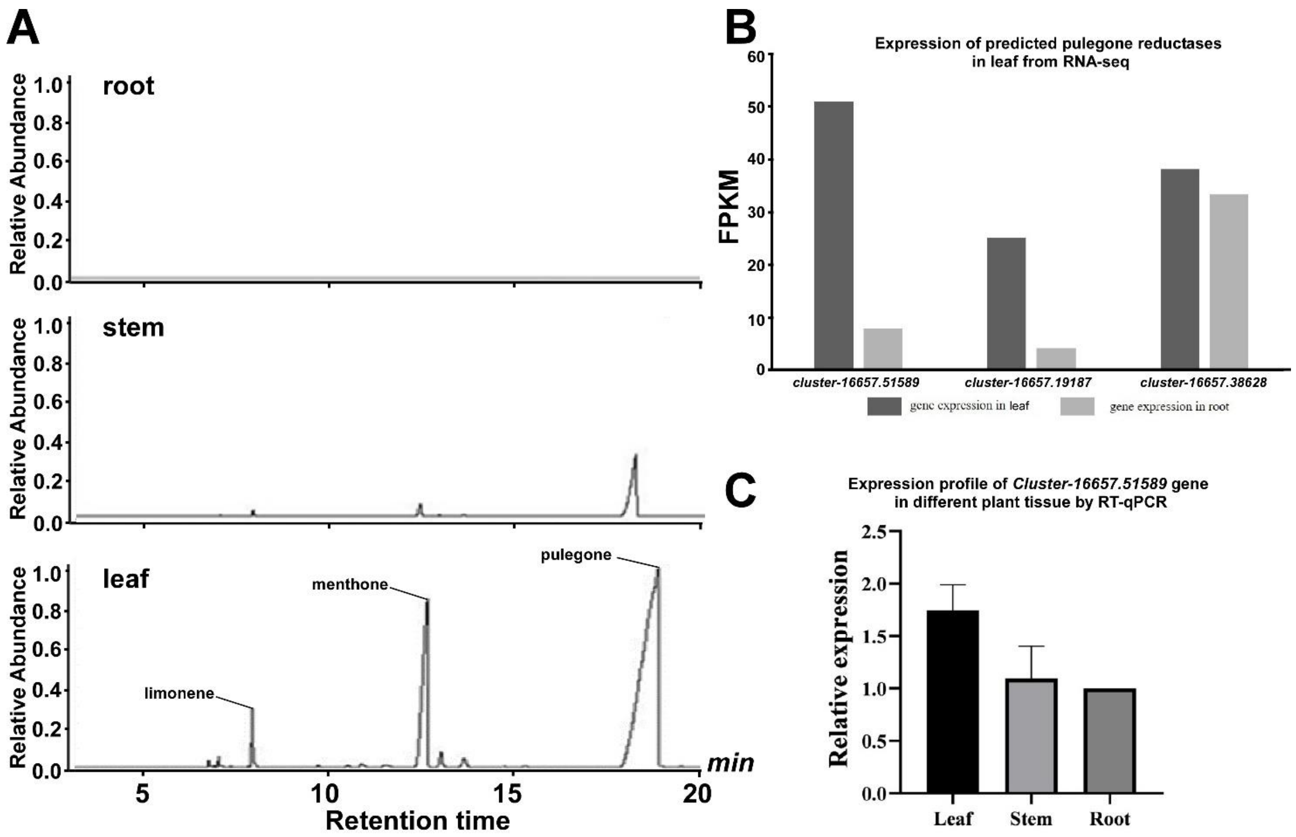

Supplementary Figure 4. Expression profile of predicted pulegone reductase genes in different tissues of *N. tenuifolia* coupled with metabolite analysis of extracts from these tissues. (A) GC results for metabolite analysis of extracts from different plant tissues (leaf, stem and root); **(B)** expression analysis of predicted pulegone reductase genes (*cluster-16657-51589*, cluster-*16657-19187*, and *cluster-16657-38628*) in shoot from RNA-seq data; FPKM, expected number of Fragments Per Kilobase of transcript sequence per Millions base pairs sequenced; **(C)** expression profile of *cluster-16657-51589* gene in different plant tissue (leaf, stem and root).

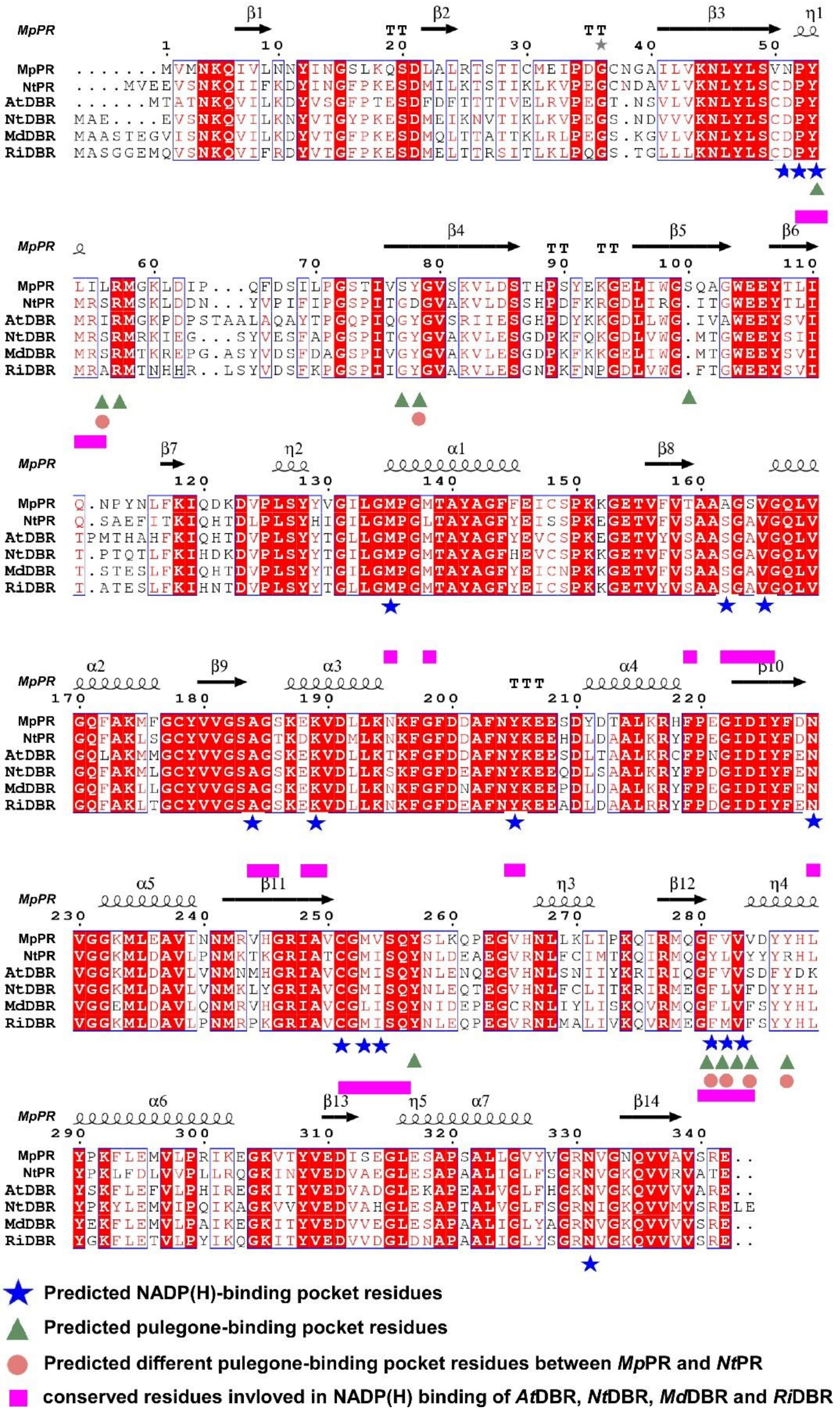

Supplementary Figure 5. Structural based sequence alignment of different DBRs. The invariant residues among MDR superfamily reductases are highlighted in red, and conserved amino acids are

boxed. predicted residues in the NADP(H) binding site of are indicated with blue five-pointed stars; predicted residues in the pulegone binding site are indicated with green triangles; predicted different pulegone-binding residues between *Mp*SDT and *Nt*SHT are indicated with red cycles. Conserved residues involved in NADP(H) binding of *At*DBR, *Nt*DBR, *Md*DBR and *Ri*DBR are indicated with purple rectangle. The secondary structure elements of *Mp*PR are shown at the top. *Mp*PR, pulegone reductase from *Mentha piperita*; *Nt*PR, pulegone reductase from *Nepeta tenuifolia*; *At*DBR, double bond reductase from *Arabidopsis thaliana*; *Nt*DBR, double bond reductase from *Nicotiana tabacum*; *Md*DBR, double bond reductase from *Malus domestica*; *Ri*DBR, double bond reductase from *Rubus idaeus*; The residue numbering is according to *At*SDT.

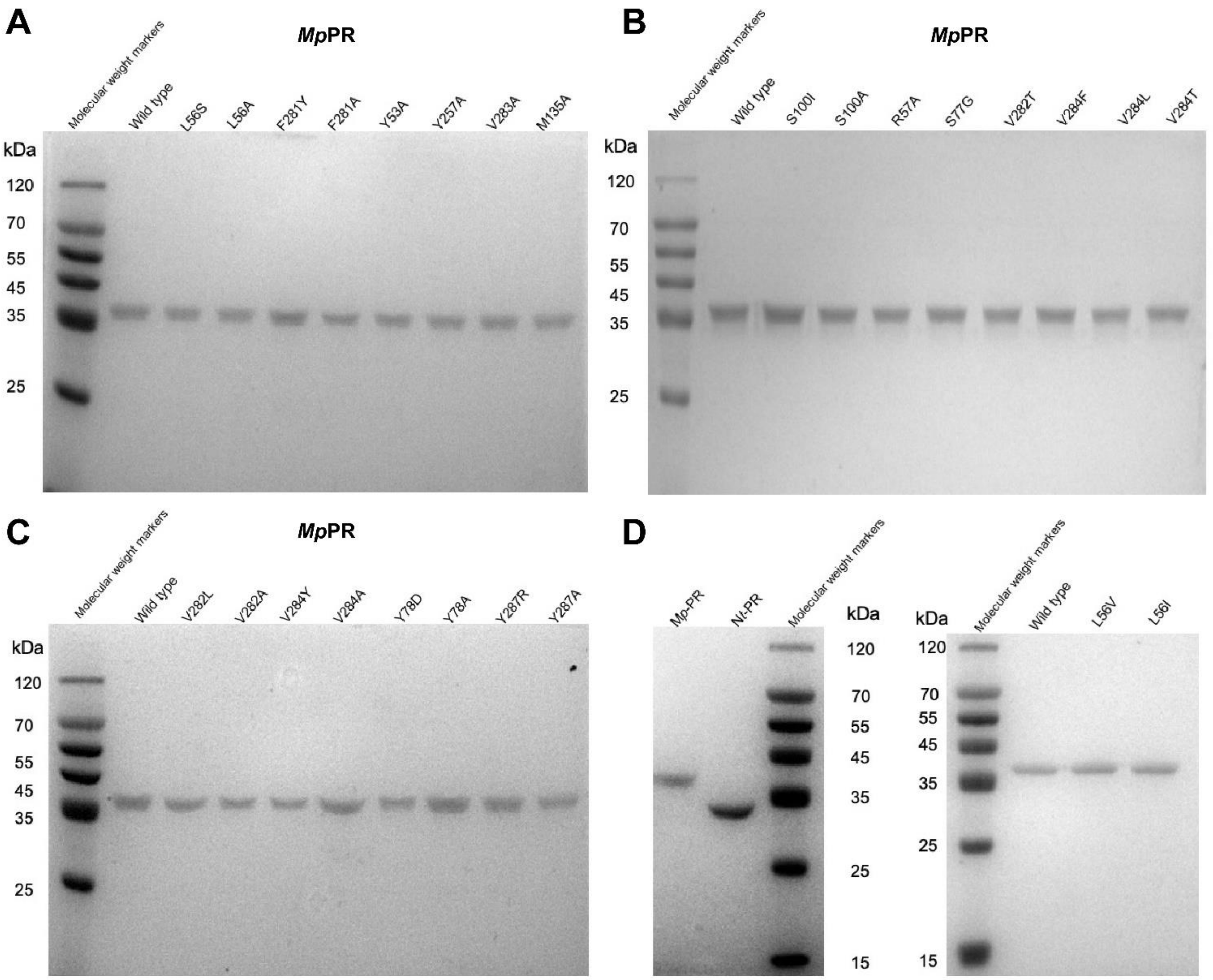

Supplementary Figure 6. SDS PAGE results for wild-type *Mp*PR, the mutants of *Mp*PR and *Nt*PR proteins.

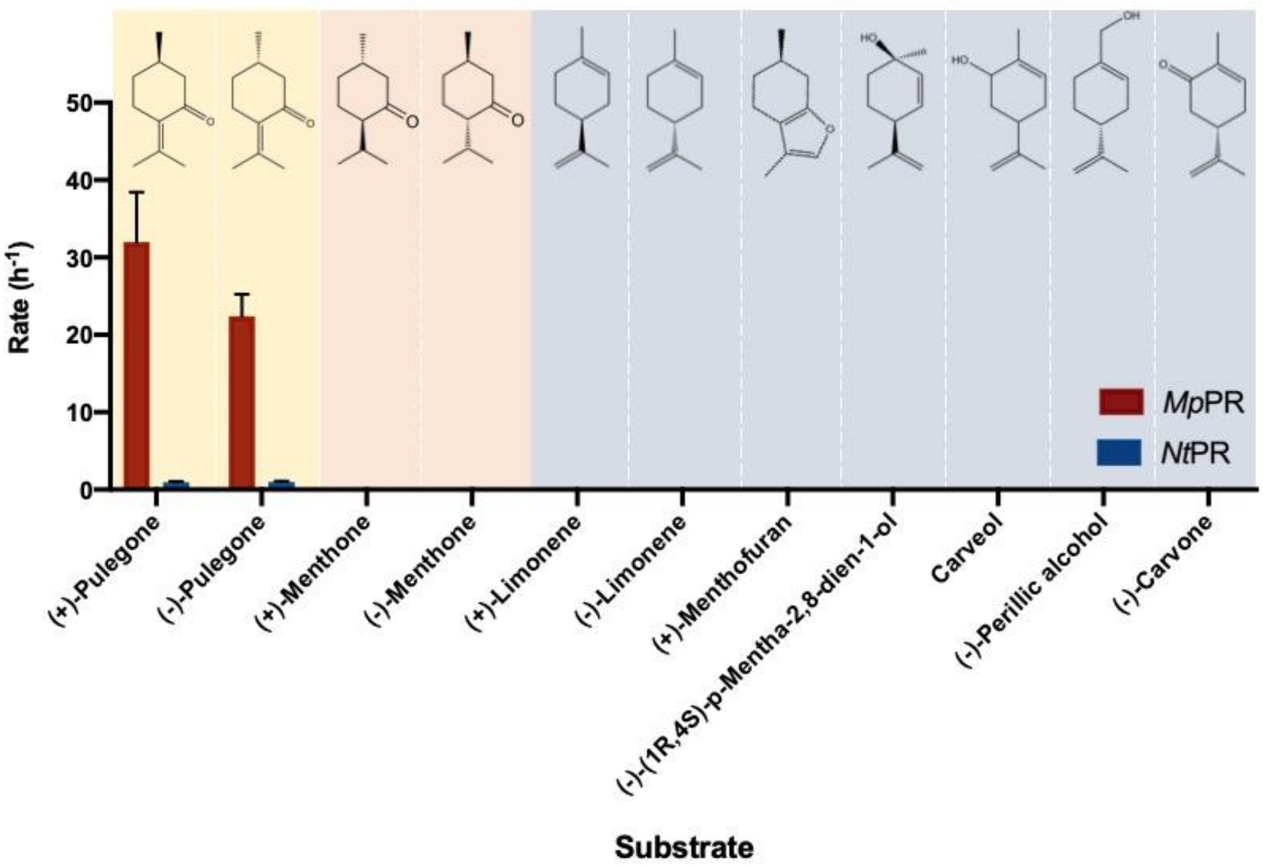

Supplementary Figure 7. Substrate selectivity of *Mp*PR and *Nt*PR by feeding with different substrates. Turnover rates of purified PR towards different substrates (20 µM) were measured. Reactions were performed as described in Materials and Methods**.**

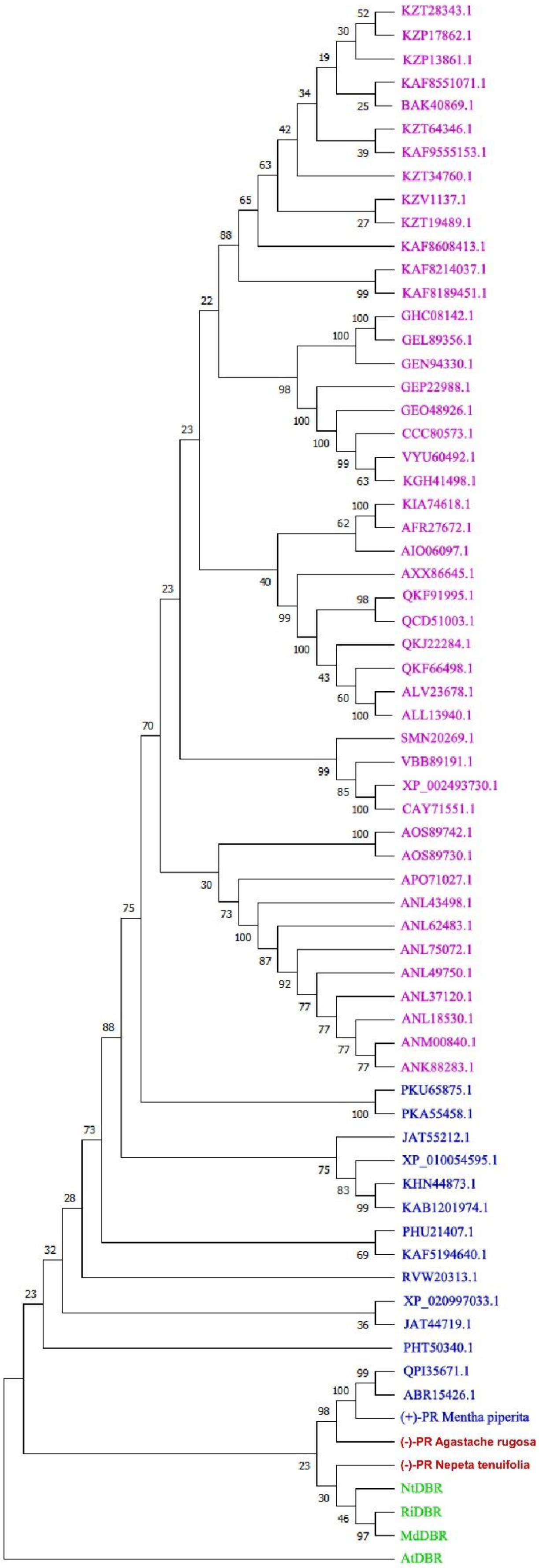
KZT28343.1
KZP17862.1
KZP13861.1
KAF8551071.1
BAK40869.1
KZT64346.1
KAF9555153.1
KZT34760.1
KZV1137.1
KZT19489.1
KAF8608413.1
KAF8214037.1
KAF8189451.1
GHC08142.1
GEL89356.1
GEN94330.1
GEP22988.1
GEO48926.1
CCC80573.1
VYU60492.1
KGH41498.1
KIA74618.1
AFR27672.1
AIO06097.1
AXX86645.1
QKF91995.1
QCD51003.1
QKJ22284.1
QKF66498.1
ALV23678.1
ALL13940.1
SMN20269.1
VBB89191.1
XP_002493730.1
CAY71551.1
AOS89742.1
AOS89730.1
APO71027.1
ANL43498.1
ANL62483.1
ANL75072.1
ANL49750.1
ANL37120.1
ANL18530.1
ANM00840.1
ANK88283.1
PKU65875.1
PKA55458.1
JAT55212.1
XP_010054595.1
KHN44873.1
KAB1201974.1
PHU21407.1
KAF5194640.1
RVW20313.1
XP_020997033.1
JAT44719.1
PHT50340.1
QPI35671.1
ABR15426.1
(+)-PR Mentha piperita
(-)-PR Agastache rugosa
(-)-PR Nepeta tenuifolia
NtDBR
RiDBR
MdDBR
AtDBR

Supplementary Figure 8. Phylogenetic analysis of alkene reductases from the MDR superfamily shown in the traditional rectangular view with branch length corresponding to the evolutionary distance.

The clades of alkene reductases from the MDR superfamily in which new identified pulegone reductase *N. tenuifolia* (-)-pulegone reductase (*Nt*PR), *A. rugose* (-)-pulegone reductase (*Ar*PR), and previous identified pulegone reductases including *M. piperita* (+)-pulegone reductase (*Mp*PR) located are colored in red and blue, respectively. The neighboring clade mainly bearing the double bond reductases, such as *Md*DBR, *At*DBR, *Nt*DBR, and *Ri*DBR from the MDR superfamily, the structures and functions of which have been previously investigated, is colored in green. The rest clade including other unrelated alkene reductases belonging to the MDR superfamily but the structures and functions of which have not been previously well studied is colored in purple. The alkene reductases IDs from the MDR superfamily mentioned above are referring to the National Center for Biotechnology Information(NCBI).

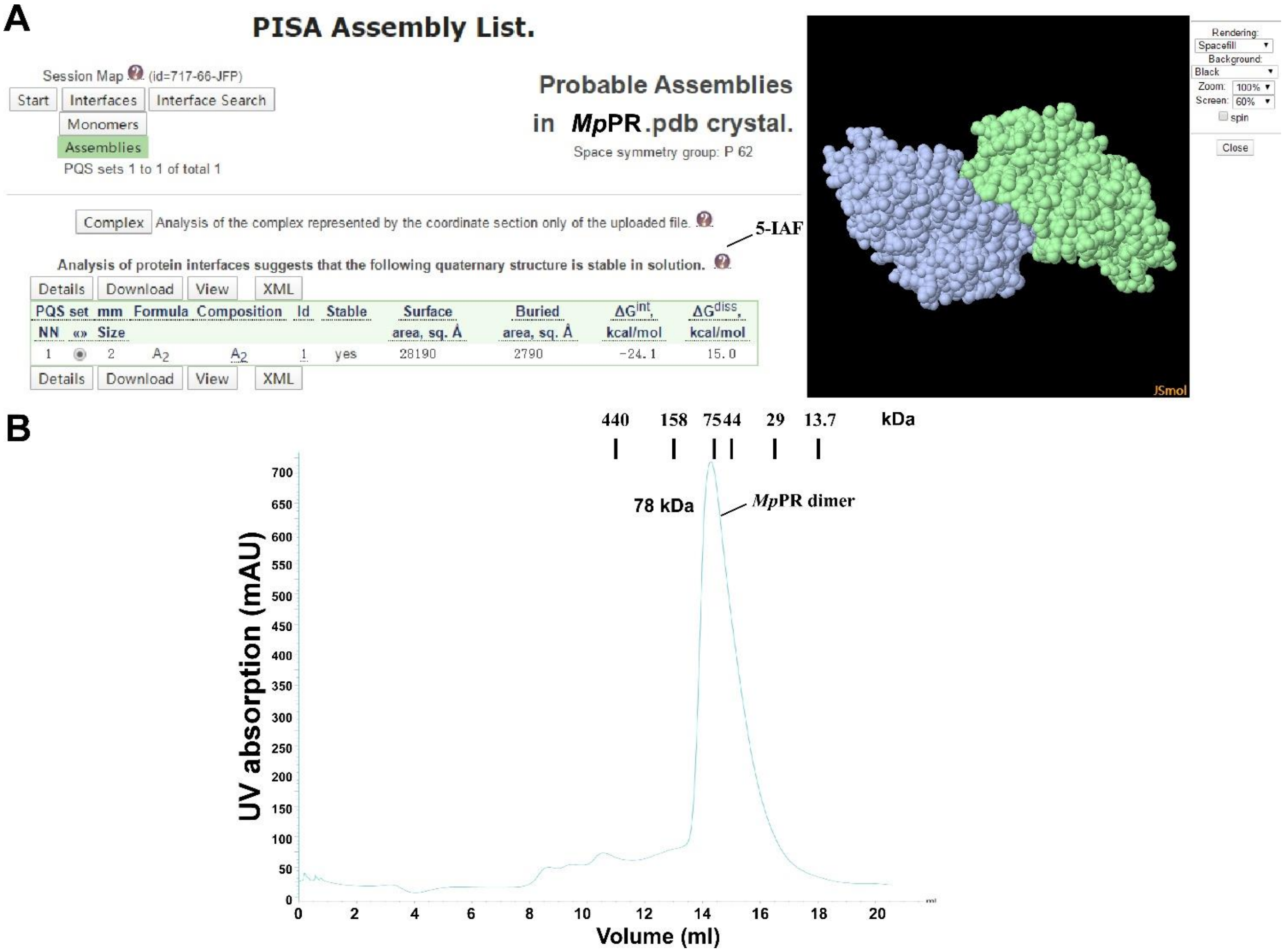

Supplementary Figure 9. The functional form of *Mp*PR is a homodimer. (A) The results of the PDBePISA server calculations **(B)** The results of gel filtration of *Mp*PR.

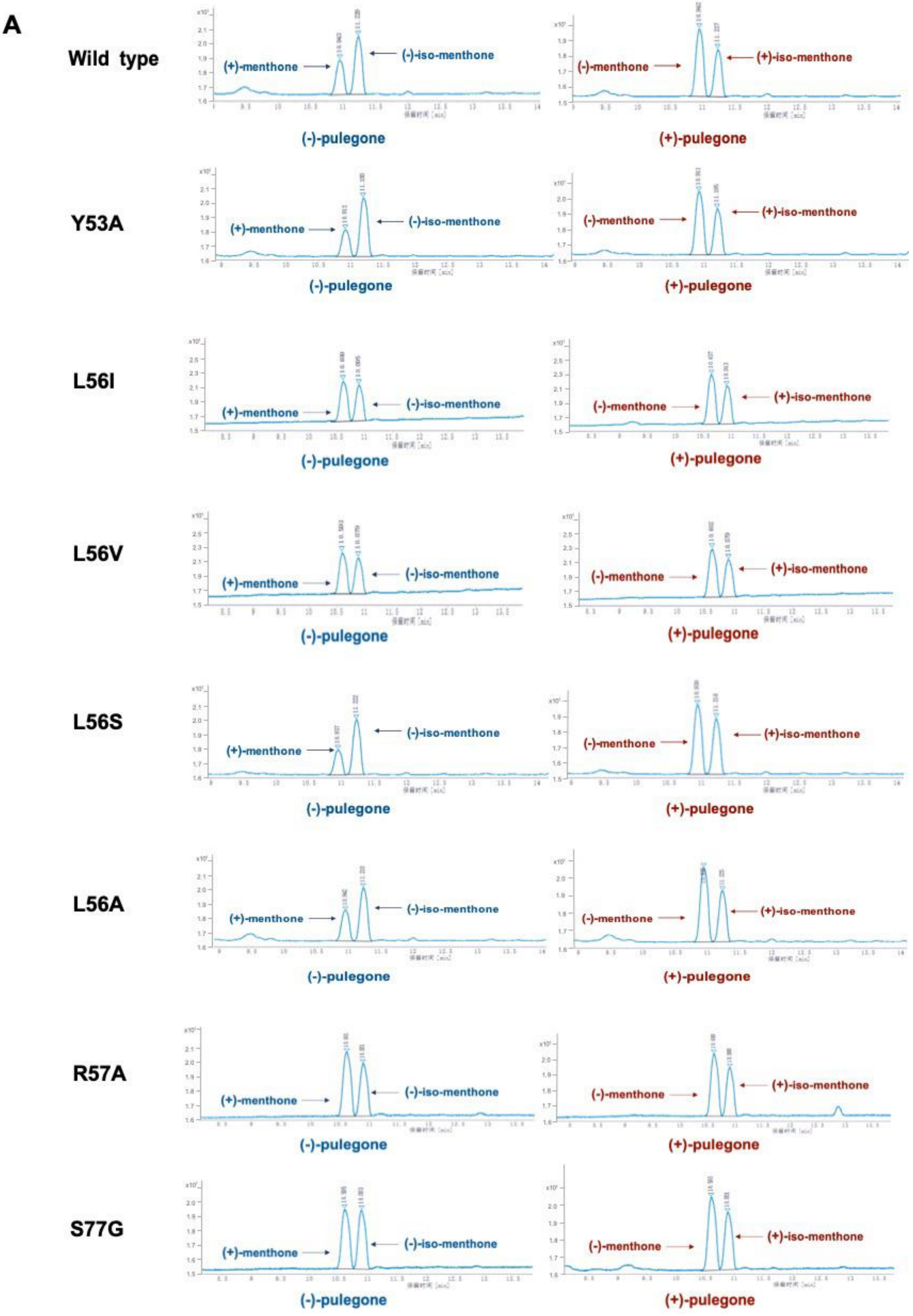
A
Wild type
Y53A
L56I
L56V
L56S
L56A
R57A
S77G
(+)-menthone
(-)-iso-menthone
(-)-menthone
(+)-iso-menthone
(-)-pulegone
(+)-pulegone

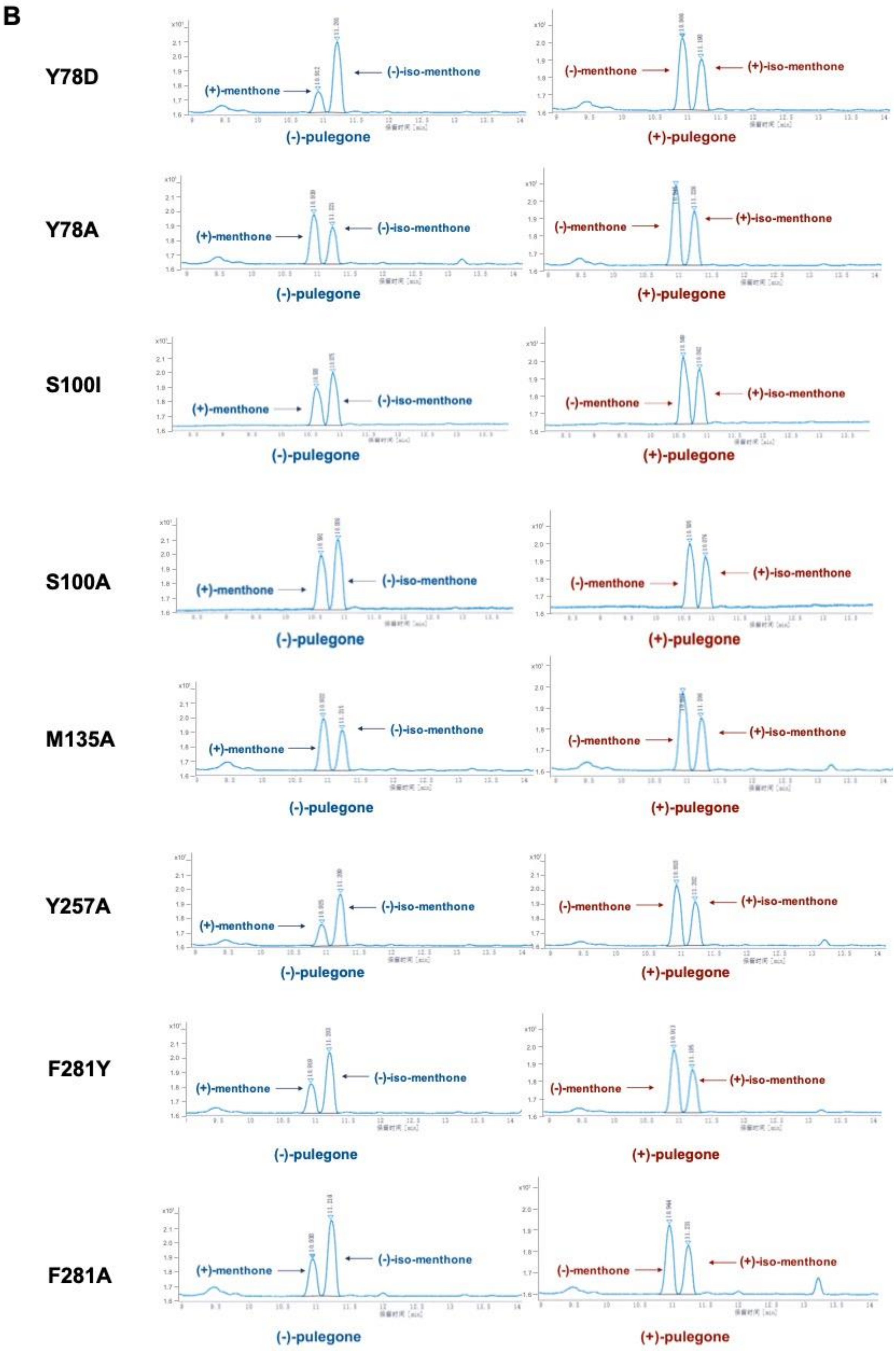
B
Y78D
(+)-menthone
(-)-iso-menthone
(-)-pulegone
(-)-menthone
(+)-iso-menthone
(+)-pulegone
Y78A
(+)-menthone
(-)-iso-menthone
(-)-pulegone
(-)-menthone
(+)-iso-menthone
(+)-pulegone
S100I
(+)-menthone
(-)-iso-menthone
(-)-pulegone
(-)-menthone
(+)-iso-menthone
(+)-pulegone
S100A
(+)-menthone
(-)-iso-menthone
(-)-pulegone
(-)-menthone
(+)-iso-menthone
(+)-pulegone
M135A
(+)-menthone
(-)-iso-menthone
(-)-pulegone
(-)-menthone
(+)-iso-menthone
(+)-pulegone
Y257A
(+)-menthone
(-)-iso-menthone
(-)-pulegone
(-)-menthone
(+)-iso-menthone
(+)-pulegone
F281Y
(+)-menthone
(-)-iso-menthone
(-)-pulegone
(-)-menthone
(+)-iso-menthone
(+)-pulegone
F281A
(+)-menthone
(-)-iso-menthone
(-)-pulegone
(-)-menthone
(+)-iso-menthone
(+)-pulegone

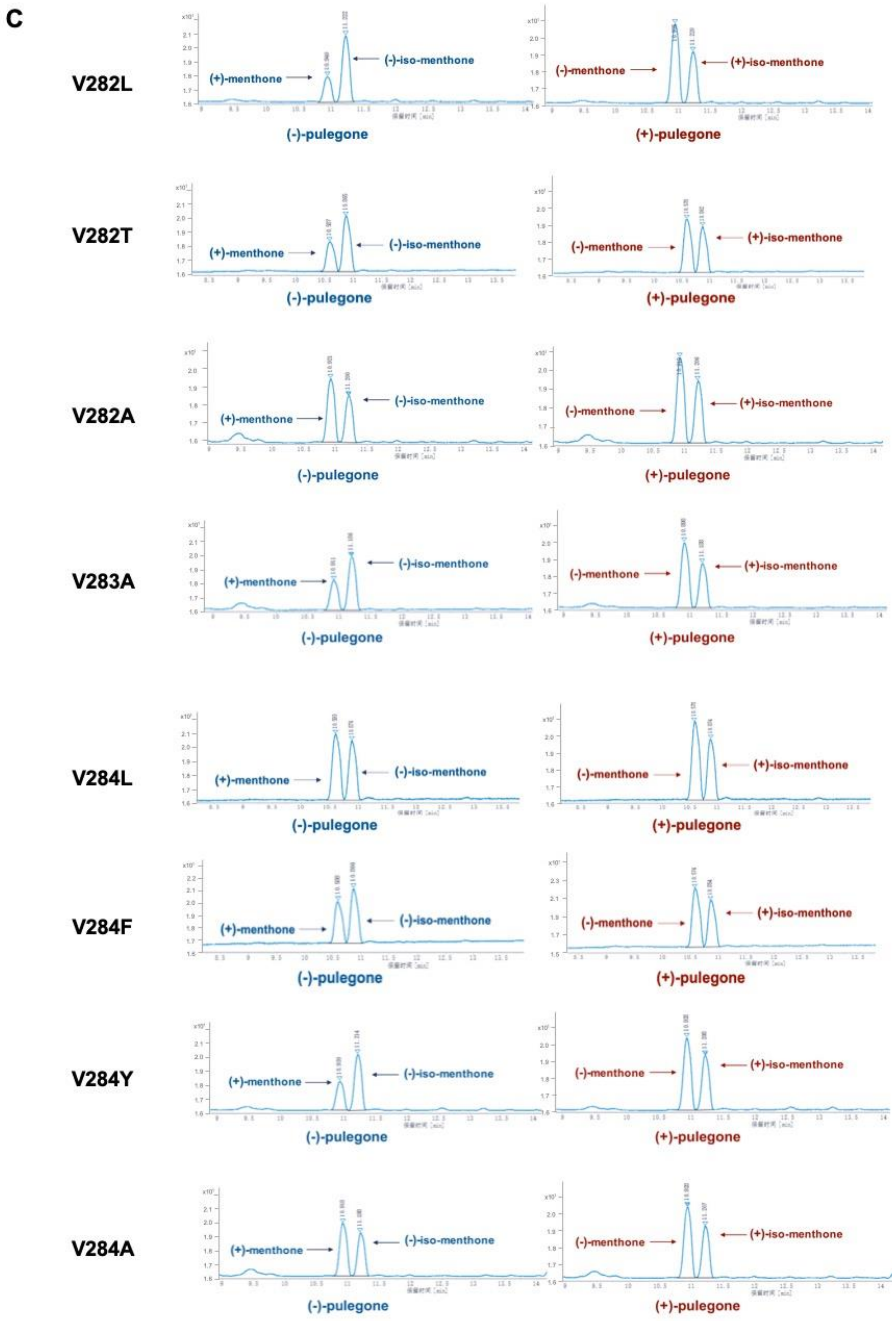
C
V282L
(+)-menthone
(-)-iso-menthone
(-)-pulegone
(-)-menthone
(+)-iso-menthone
(+)-pulegone
V282T
(+)-menthone
(-)-iso-menthone
(-)-pulegone
(-)-menthone
(+)-iso-menthone
(+)-pulegone
V282A
(+)-menthone
(-)-iso-menthone
(-)-pulegone
(-)-menthone
(+)-iso-menthone
(+)-pulegone
V283A
(+)-menthone
(-)-iso-menthone
(-)-pulegone
(-)-menthone
(+)-iso-menthone
(+)-pulegone
V284L
(+)-menthone
(-)-iso-menthone
(-)-pulegone
(-)-menthone
(+)-iso-menthone
(+)-pulegone
V284F
(+)-menthone
(-)-iso-menthone
(-)-pulegone
(-)-menthone
(+)-iso-menthone
(+)-pulegone
V284Y
(+)-menthone
(-)-iso-menthone
(-)-pulegone
(-)-menthone
(+)-iso-menthone
(+)-pulegone
V284A
(+)-menthone
(-)-iso-menthone
(-)-pulegone
(-)-menthone
(+)-iso-menthone
(+)-pulegone

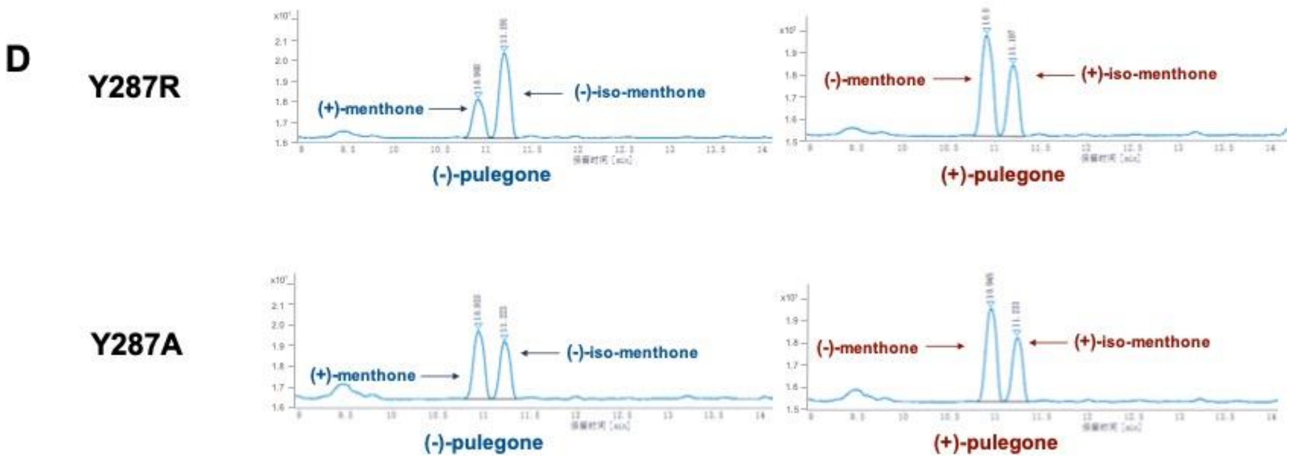

Supplementary Figure 10. GC profiles show the catalytic activity of wild-type *Mp*PR and its mutants using the substrate (+)-pulegone or (-)-pulegone. (A) (B) (C) (D) Left panel, GC profiles of the products generated by the reactions between *Mp*PR and (-)-pulegone; Right panel, GC profiles of the products generated by the reactions between *Mp*PR and (+)-pulegone. The horizontal axis represents retention time and the vertical axis represents relative abundance.

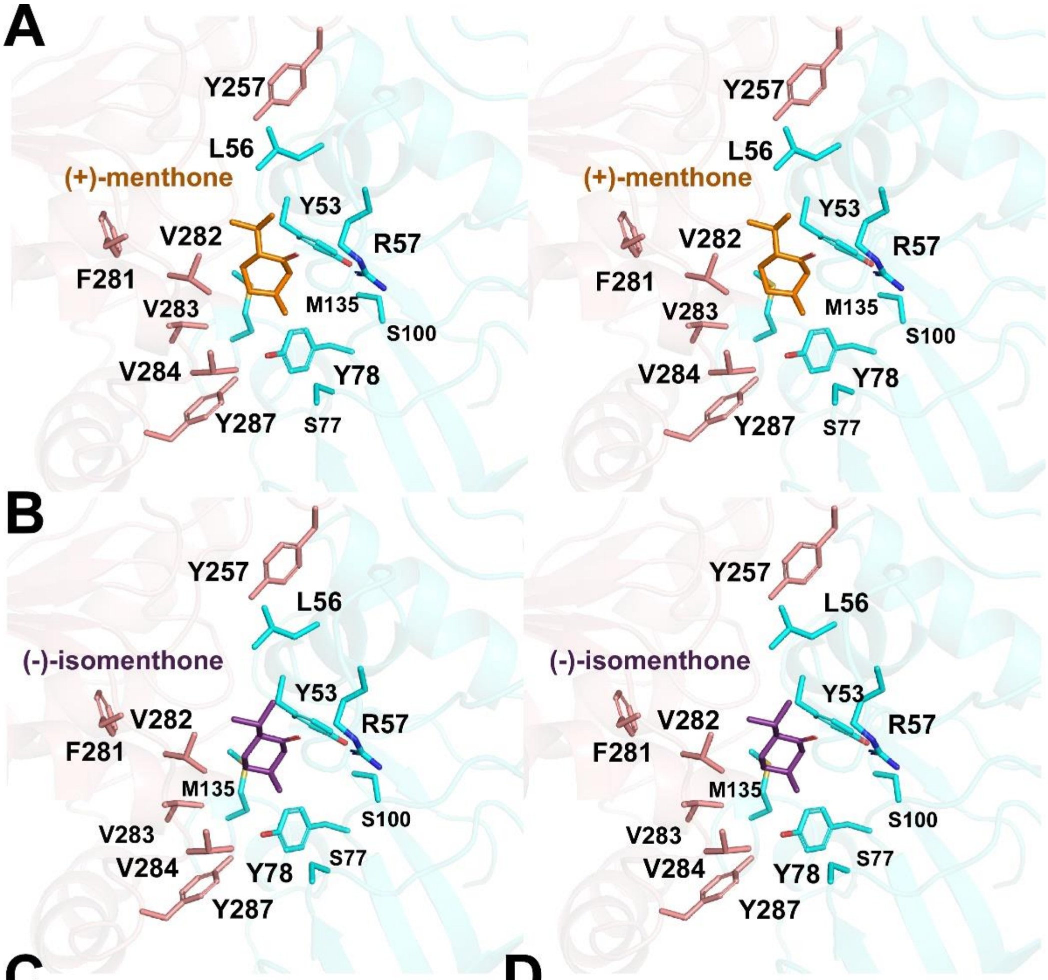

C

Contribution	(+)-menthone	(-)-isomenthone
△G_{VDW}[a]	-84.55	-77.84
△G_{ele}[b]	-108.65	-73.80
△G_{GB}[c]	107.19	85.22
△G_{GA}[d]	-48.22	-43.98
△G_{bind}[e]	-141.26	-116.74

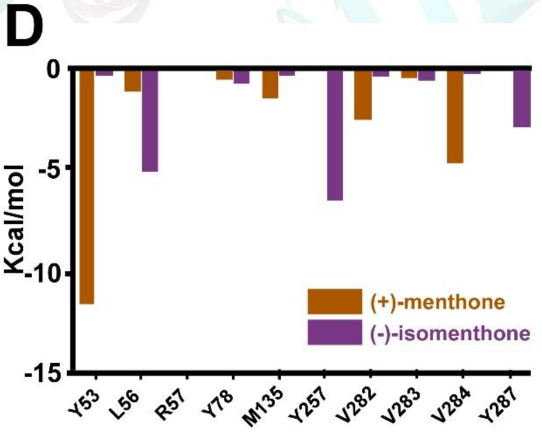

Supplementary Figure 11. Structural docking results and predicted binding parameters of *Mentha piperita* (+)-pulegone reductase (*Mp*PR) with the products (+)-menthone or (-)-isomenthone by MD simulations. (A) Stereo view shows the key residues of *Mp*PR interacting with the product (+)-menthone. **(B)** Stereo view shows the key residues of *Mp*PR interacting with the product (-)-isomenthone. **(C)** Calculated intermolecular forces and free binding energies between (+)-menthone or (-)-isomenthone by MD simulations. The parameters are the same as **Figure 5C**. **(D)** Strengths of hydrophobic interactions contributed by the binding pocket residues of *Mp*PR predicted by MD simulations.

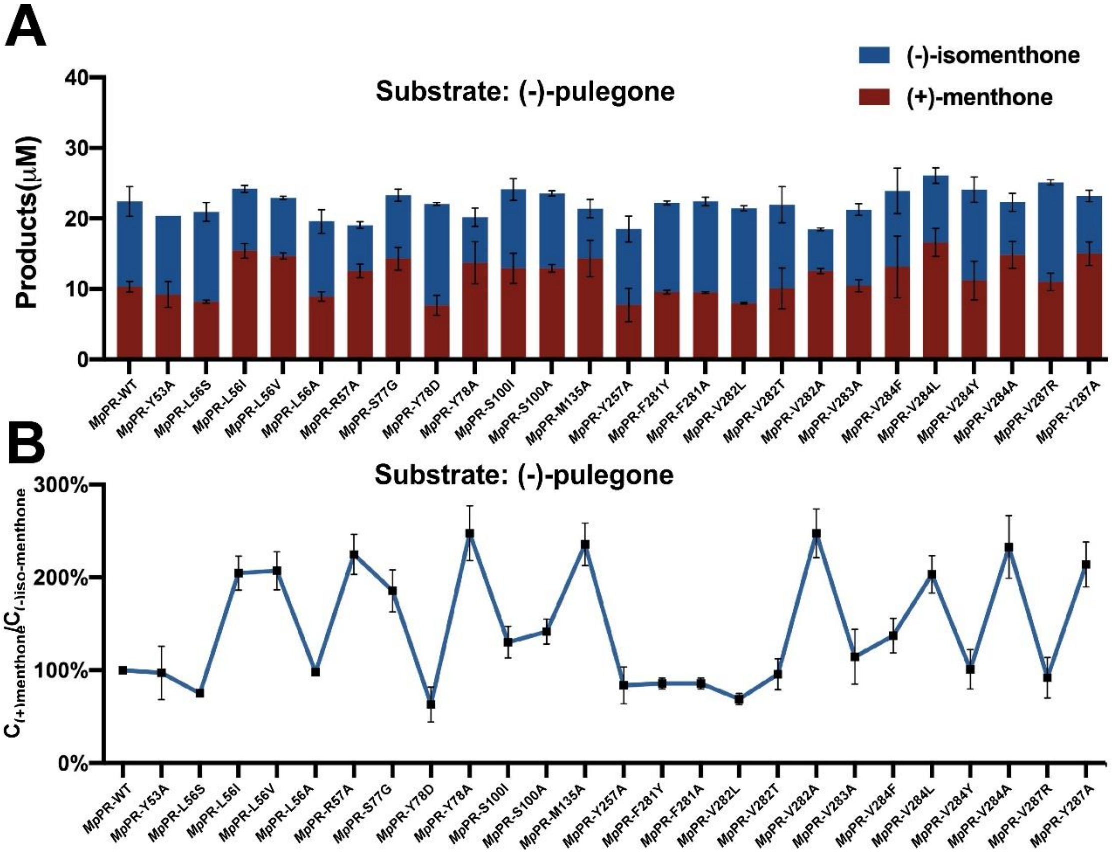

Supplementary Figure 12. GC-MS results for wild-type *Mp*PR and its mutants. (A) Quantification of products (+)-menthone and (-)-iso-menthone from substrate (-)-pulegone for wild-type *Mp*PR or its mutants. Reactions(0.4mL) were performed in buffer (50 mM KH_2PO_4, 10 % sorbitol, 1 mM DTT, pH 7.5) containing 20uM substrate,10mM NADPH tetrasodium salt hydrate, 6 mM glucose-6-phosphate, 20 U glucose-6-phosphate dehydrogenase and 30μM *Mp*PR. 0.2 mL of n-hexane was added on the top of the reaction solution. Reaction was carried out at 31°C for 1h. The concentration of products in n-hexane was determined by gas chromatography. Wild-type *Mp*PR and its mutants react under the same conditions. **(B)** The percentage ratio of the products (+)-menthone and (-)-isomenthone converted from Fig. S12 A. Wild type's ratio of (+)-menthone and (-)-isomenthone is normalized to 100%. Data are presented as mean ± S.D. of triplicate experiments.

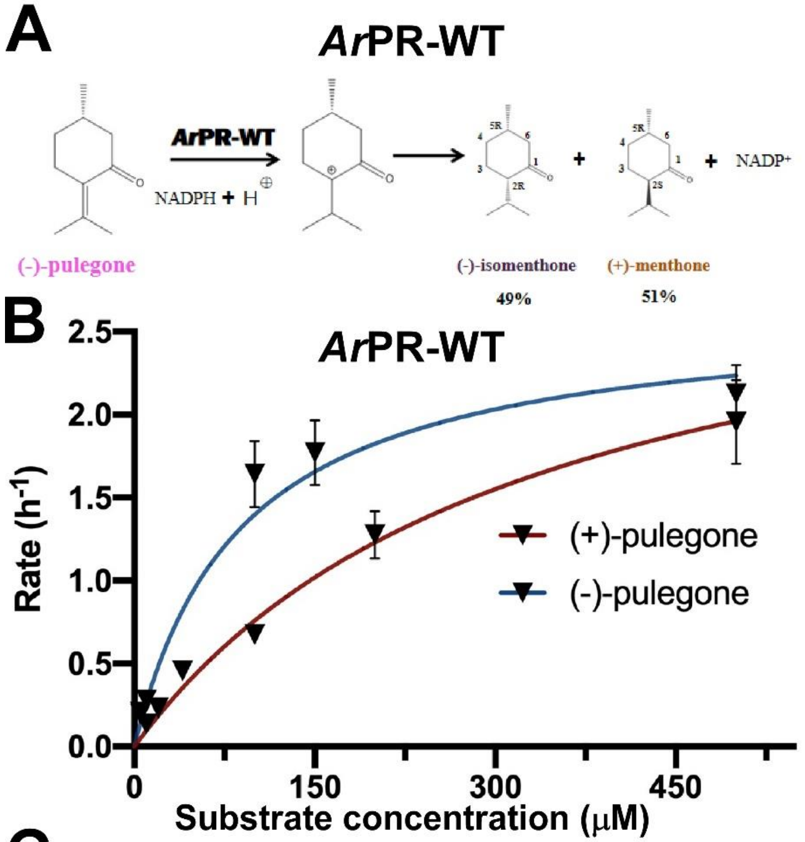

C

*Ar*PR-WT	(+)-pulegone		(-)-pulegone	
	K_m(μM)	V_{max}(μM/h)	K_m(μM)	V_{max}(μM/h)
	326.85 ± 41.75	3.26 ± 0.47	88.64 ± 4.39	2.63 ± 0.15

Supplementary Figure 13. Functional characterization of (-)-pulegone reductase from *A. rugosa*. **(A)** (-)-Pulegone reductase from *A. rugosa* reduces the (-)-pulegone to (+)-menthone by using NADPH. **(B)** Michaelis-Menten equation fitting curve for (-)-pulegone reductase from *A. rugosa*. **(C)** Kinetic Parameters Measurement for wild-type (-)-pulegone reductase from *A. rugosa*. **Supplementary table1.** provides enzyme concentration and substrate concentration for each reaction.

Supplementary table 1. Parameters for enzyme kinetics assays for the wild-type *Mp*PR and its mutants. And parameters for enzyme kinetics assays for the *Nt*PR and *Ar*PR.

Protein	Amount of protein (μM)	Substrate concentration range (μM)	Reaction time
***Mp*PR**	**0.06**	**2 - 50**	**1 h**
***Nt*PR**	**19.69**	**5 - 500**	**16 h**
***Ar*PR**	**5.88**	**5 - 500**	**16 h**
***Mp*PR-L56S**	**3.04**	**40 - 1000**	**1 h**
***Mp*PR-V282L**	**0.30**	**5 - 120**	**1 h**
***Mp*PR-V284Y**	**0.30**	**5 - 120**	**1 h**

Supplementary table 2. Primers used for qRT-PCR

Gene	Sequence (5’-3’)
NtEF-1α	**F: GACAAGCCTCTTCGTCTCCC**<br>**R: GTTCGATGCAACAAACCCAC**
NtPR	**F: GAGGAAGTGAGCAACAAACAGA**<br>**R: GGATCGCACGACAAGTAGAGAT**

Supplementary table 3. Source species for the alkene reductases from the MDR superfamily used in the phylogenetic analysis.

Alkene reductases Protein	Source species
ANM00840.1	*Rhizobium phaseoli*
ANL75072.1	*Rhizobium phaseoli*
ANL49750.1	*Rhizobium phaseoli*
ANL37120.1	*Rhizobium phaseoli*
ANL18530.1	*Rhizobium* sp. N1314
ANK88283.1	*Rhizobium* sp. N731
APO71027.1	*Rhizobium gallicum*
KZV71137.1	*Peniophora* sp. CONT
ANL62483.1	*Rhizobium phaseoli*
KAF9555153.1	*Agrocybe pediades*
KAF8608413.1	*Ceratobasidium* sp. AG-I

KAF8551071.1	*Xerocomus badius*
KAF8214037.1	*Mycena galopus*
KAF8189451.1	*Mycena galopus*
ANL43498.1	*Rhizobium phaseoli*
KZT64346.1	*Daedalea quercina L*
KZT34760.1	*Sistotremastrum suecicum*
KZT28343.1	*Neolentinus lepideus*
KZT19489.1	*Neolentinus lepideus*
KZP17862.1	*Fibularhizoctonia*
KZP13861.1	*Fibularhizoctonia*
BAK40869.1	*Phanerochaete sordida*
GHC08142.1	*Pediococcus parvulus*
GEL89356.1	*Pediococcus parvulus*
GEO48926.1	*Lactiplantibacillus pentosus*
GEP22988.1	*Lentilactobacillus diolivorans*
GEN94330.1	*Pediococcus ethanolidurans*
VTU60492.1	*Lactobacillus plantarum*
AOS89730.1	*Aspergillus niger*
CCC80573.1	*Lactobacillus plantarum*
KGH41498.1	*Lactobacillus plantarum*
AIO06097.1	*Thauera aromatica*

QKJ22284.1	*Poseidonibacter lekithochrous*
QKF91995.1	*Campylobacter* sp.
QKF66498.1	*Arcobacter venerupis*
QCD51003.1	*Campylobacter* sp.
AXX86645.1	*Malaciobacter marinus*
VBB89191.1	*Yarrowia lipolytica*
XP_002493730.1	*Komagataella phaffii*
SMN20269.1	*Kazachstania saulgeensis*
AOS89742.1	*Aspergillus welwitschiae*
ALV23678.1	*Campylobacter iguaniorum*
AII13940.1	*Campylobacter iguaniorum*
CAY71551.1	*Komagataella phaffii*
KIA74618.1	*Arthrobacter* sp. MWB30
AFR27672.1	*Arthrobacter* sp. Rue61a
(+)-PR	*Mentha x piperita*
XP_010054595.1	*Eucalyptus grandis*
ABR15426.1	*Mentha canadensis*
KAB1201974.1	*Morella rubra*
PKU65875.1	*Dendrobium catenatum*
PHU21407.1	*Capsicum chinense*
PHT50340.1	*Capsicum baccatum*

KHN44873.1	*Glycine soja*
QPI35671.1	*Mentha canadensis*
KAF5194640.1	*Thalictrum thalictroides*
XP_020997033.1	*Arachis duranensis*
JAT44719.1	*Anthurium amnicola*
JAT55212.1	*Anthurium amnicola*
PKA55458.1	*Apostasia shenzhenica*
RVW20313.1	*Vitis vinifera*
*At*DBR	*Arabidopsis thaliana*
*Nt*DBR	*Nicotiana tabacum*
*Ri*DBR	*Rubus idaeus*
*Md*DBR	*Malus domestica* L.

Kumar, S., Stecher, G., Li, M., Knyaz, C., and Tamura, K. (2018). MEGA X: Molecular Evolutionary Genetics Analysis across Computing Platforms. *Mol Biol Evol* 35**,** 1547-1549.

Tamura, K., Dudley, J., Nei, M., and Kumar, S. (2007). MEGA4: Molecular Evolutionary Genetics Analysis (MEGA) software version 4.0. *Mol Biol Evol* 24**,** 1596-1599.

Tamura, K., and Nei, M. (1993). Estimation of the number of nucleotide substitutions in the control region of mitochondrial DNA in humans and chimpanzees. *Mol Biol Evol* 10**,** 512-526.

Tamura, K., Stecher, G., Peterson, D., Filipski, A., and Kumar, S. (2013). MEGA6: Molecular Evolutionary Genetics Analysis version 6.0. *Mol Biol Evol* 30**,** 2725-2729.
